# Motion-compensated T_1_ mapping in cardiovascular magnetic resonance imaging: a technical review

**DOI:** 10.3389/fcvm.2023.1160183

**Published:** 2023-09-08

**Authors:** Calder D. Sheagren, Tianle Cao, Jaykumar H. Patel, Zihao Chen, Hsu-Lei Lee, Nan Wang, Anthony G. Christodoulou, Graham A. Wright

**Affiliations:** ^1^ Department of Medical Biophysics, University of Toronto, Toronto, ON, Canada; ^2^ Sunnybrook Research Institute, Sunnybrook Health Sciences Centre, Toronto, ON, Canada; ^3^ Biomedical Imaging Research Institute, Cedars-Sinai Medical Center, Los Angeles, CA, United States; ^4^ Department of Bioengineering, University of California, Los Angeles, CA, United States; ^5^ Department of Radiology, Stanford University, Stanford, CA, United States

**Keywords:** *T*_1_ mapping, motion-corrected MRI, motion-resolved MRI, cardiovascular MRI, multiparametric MRI

## Abstract

$T_{1}$ mapping is becoming a staple magnetic resonance imaging method for diagnosing myocardial diseases such as ischemic cardiomyopathy, hypertrophic cardiomyopathy, myocarditis, and more. Clinically, most $T_{1}$ mapping sequences acquire a single slice at a single cardiac phase across a 10 to 15-heartbeat breath-hold, with one to three slices acquired in total. This leaves opportunities for improving patient comfort and information density by acquiring data across multiple cardiac phases in free-running acquisitions and across multiple respiratory phases in free-breathing acquisitions. Scanning in the presence of cardiac and respiratory motion requires more complex motion characterization and compensation. Most clinical mapping sequences use 2D single-slice acquisitions; however newer techniques allow for motion-compensated reconstructions in three dimensions and beyond. To further address confounding factors and improve measurement accuracy, $T_{1}$ maps can be acquired jointly with other quantitative parameters such as $T_{2}$, $T_{2}^{*}$, fat fraction, and more. These multiparametric acquisitions allow for constrained reconstruction approaches that isolate contributions to $T_{1}$ from other motion and relaxation mechanisms. In this review, we examine the state of the literature in motion-corrected and motion-resolved $T_{1}$ mapping, with potential future directions for further technical development and clinical translation.

## 1. Introduction

### 1.1. Clinical applications of $T_{1}$ mapping

The nuclear magnetic resonance tissue parameter $T_{1}$ is sensitive to inflammation and fibrosis in the heart (1). $T_{1}$-weighted ($T_{1}w$) magnetic resonance imaging (MRI), both pre- and post-contrast administration, is thus highly prevalent to diagnose these conditions. Quantitative $T_{1}$ mapping can add sensitivity to subtle and diffuse pathological changes by directly measuring the underlying tissue parameters (2, 3). The objective nature of quantitative mapping is also promising for early diagnosis and longitudinal follow-up. $T_{1}$ mapping is used clinically to standardize images across larger patient populations and further refine disease stratification. Clinically, the 2017 SCMR and EACVI recommendations paper endorse cardiac parametric mapping, including $T_{1}$ mapping, for diagnosis and clinical management of diseases such as iron overload, cardiac amyloidosis, myocarditis, and heart failure (4).

$T_{1}$ mapping can be used as a clinical diagnosis tool for focal and diffuse myocardial diseases. Focal diseases can be determined by the average $T_{1}$ value within a region of interest around the lesion, and diffuse diseases can be determined by variations in global average $T_{1}$ from the institutional reference values. However, the reliability of measurements around the left ventricular lateral wall could be compromised by the lung-tissue interface and the associated changes in magnetic susceptibility. It has hence been proposed to utilize septal regions only for diseases with diffuse myocardial involvement, which has shown better reproducibility and minimizes the inter- and intra-observer differences (5).

$T_{1}$ mapping has demonstrated clinical utility in the diagnosis and monitoring of nonischemic and ischemic cardiomyopathies. Nonischemic cardiomyopathy results in myocardial impairment and remodelling due to diffuse and subtle myocardial changes that are hard to distinguish from normal variation, limiting early detection and effective management. As shown in a multi-center study, native $T_{1}$ values alone were predictive of the all-cause mortality and heart failure endpoint in nonischemic cardiomyopathy patients (6). Another meta-analysis has also found a significant difference in $T_{1}$ between nonischemic cardiomyopathy patients and healthy controls, supporting the clinical potential of $T_{1}$ mapping in this patient cohort (7).

Post-contrast $T_{1}w$ late gadolinium enhancement (LGE) imaging has been used to characterize regions of dense scar and heterogeneous fibrosis in patients with ischemic cardiomyopathy to plan ventricular ablation procedures (8). Post-contrast $T_{1}$ mapping has shown promise for robustly identifying arrhythmogenic tissue in post-myocardial infarction patients (9, 10). Recently, native and post-contrast $T_{1}$ mapping has been used to characterize diffuse fibrosis in patients with hypertrophic cardiomyopathy and assess their risk for arrhythmias and sudden cardiac death (11–14). Further, two recent studies showed that native $T_{1}$ and extracellular volume (ECV) values are independent predictors of sudden cardiac death and arrhythmia recurrence in patients with dilated cardiomyopathy (15, 16). Native $T_{1}$ and ECV mapping is also useful in the diagnosis of nonischemic cardiomyopathies such as: Fabry’s disease (decreased native $T_{1}$), iron overload (decreased native $T_{1}$), and cardiac amyloidosis (increased native $T_{1}$, increased ECV) (4).

### 1.2. Current clinical mapping approaches: confounding factors and limitations

The performance and reliability of quantitative methods can be evaluated using three metrics: accuracy, precision, and reproducibility. Accuracy reflects the systematic errors which remain the same given the same setting, whereas precision describes the errors due to random variations within a scan. From a statistical perspective, an inaccurate method has a high bias, whereas an imprecise method has a high variance. Reproducibility refers to inter-scan variability. In this section, we will examine different sources of error and evaluate their impacts on $T_{1}$ mapping quality.

#### 1.2.1. Cardiac and respiratory motion

Every cardiac MRI image is impacted by physiological motion inherent to the heart and observed by the heart due to respiration. When performing multi-shot $T_{1}$ mapping, parametric errors can be introduced as a result of tissue movement during and between $T_{1}w$ image acquisitions. Changes in cardiac rhythm affect the delay time after the preparation pulse, which reduces effective sampling of $T_{1}$ relaxation and may result in a loss of $T_{1}$ accuracy and precision. Abnormal heart rhythms can also affect $T_{1}$ precision and reproducibility due to varying RR interval lengths that affect $T_{1}w$ image timing. Similarly, respiratory motion affects $T_{1}$ accuracy due to motion between $T_{1}w$ images that changes the signal intensity at a given voxel. Clinical mapping sequences attempt to mitigate these artifacts by using electrocardiogram (ECG) triggering within a 10–15 heartbeat breath-hold per 2D slice, but this may fail in patients with abnormal heart rhythms and patients who cannot perform multiple consistent breath-holds in series. Even in routine clinical use, ECG triggering purposefully discards clinically valuable information provided by images of cardiac motion, e.g. ejection fraction, which must instead be collected in separate cine scans. Additionally, even in subjects who can hold their breath properly, breath-hold durations put a ceiling on the acquisition time, signal to noise ratio (SNR), and resolution of the $T_{1}$ maps. As such, $T_{1}$ mapping methods that do not require ECG gating or breath-holds have the potential to improve clinical workflow and allow for richer datasets to be collected.

#### 1.2.2. Magnetic field inhomogeneities and precession frequency offsets

Systemic imperfections such as magnetic field inhomogeneities can be major sources of error. Inhomogeneities in the static magnetic field $B_{0}$ can cause strong banding artifacts and $T_{1}$ estimation error in balanced steady-state free precession (bSSFP)-based sequences (17). Similarly, the appearance of fat in myocardial tissue results in multiple precession frequencies in a single voxel, causing $T_{1}$ estimation errors (18). $B_{0}$-related artifacts often occur in regions of tissue boundaries that cause strong susceptibility gradients that affect image quality and SNR. Imperfections in the transmit field $B_{1}$ are another error source affecting the accuracy and reproducibility of $T_{1}$ estimation; most of the existing techniques based on bSSFP or gradient echo (GRE) are sensitive to $B_{1}$ transmission inhomogeneity. It has been demonstrated that $B_{1}$-related $T_{1}$ errors tend to increase with increased flip angles (19). $B_{0}$ and $B_{1}$-related errors can occur near regions of metallic implants due to the local changes in precession frequency.

#### 1.2.3. $T_{2}$ effects and magnetization transfer

Another major source of error comes from the interaction between sequence design and other intrinsic tissue parameters: for example, the apparent $T_{1}$ of bSSFP sequences is affected by a tissue’s transverse recovery time $T_{2}$, leading to $T_{2}$-dependent errors. $T_{2}$ dependency can be introduced via the choice of magnetization preparation method or number of free parameters in the $T_{1}$ model fit. Magnetization transfer, i.e. the exchange of energy between bound water molecules and free water molecules, can also be a confounding factor to $T_{1}$ mapping and cause underestimations of reported $T_{1}$ (20). However, magnetization transfer improves the sensitivity of conventional $T_{1}$ mapping methods to cardiac fibrosis, which increases clinical utility at the cost of confounding accurate physiological measurements.

#### 1.2.4. Intra-scan precision

The aforementioned confounding factors primarily affect the accuracy and/or reproducibility of $T_{1}$ mapping. That is to say, the error or bias caused from these factors can be accounted for when these factors are characterized. On the other hand, random errors due to noise, categorized as intra-scan precision, also affects the utility of $T_{1}$ mapping methods. A common metric to measure precision is the standard deviation of $T_{1}$ values of each voxel over multiple repeats under the same settings. This metric is highly dependent on the SNR of the raw images as well as the robustness of the $T_{1}$ mapping models against noise, but is susceptible to registration errors or confounders that impact repeatability. Another metric is the standard deviation of $T_{1}$ values amongst voxels within the same myocardial segment. However, it is only appropriate to measure this in healthy volunteers, as this metric will also be impacted by focal lesions that may be present in patients.

#### 1.2.5. Inter-scan precision and reproducibility

Separate to the intra-scan precision from random noise, inter-scan precision (or reproducibility) is also very important for $T_{1}$ mapping utility. For example, although some biases of $T_{1}$ measurements can be corrected for when the confounding factors are known, it is often difficult to accurately characterize those confounding factors for varied subjects and varied systems over the long time scale needed for serial measurements. To reduce such errors, techniques that are either less sensitive to the confounding factors, or able to accurately map out the confounding factors, are beneficial. In clinical practice, precision and reproducibility can be more valuable than accuracy because precision yields consistency of measures among different type of tissues (e.g., healthy or pathological) for a certain $T_{1}$ mapping method, which facilitates horizontal and longitudinal comparisons. Another challenge for reproducibility in longitudinal cardiac MRI studies in particular is the difficulty of repeatedly localizing the same oblique planes such as short axis and four-, three-, and two chamber views in such a way that $T_{1}$ measurements over time can be reliably localized to the same tissue and tracked over time.

### 1.3. Main ideas

In this review, we examine motion-informed $T_{1}$ mapping methods that can improve patient comfort and maximize information density. Information-dense sequences spend a large percentage of the scan time acquiring unique data in various cardiac motion, respiratory motion, and $T_{1}$ contrast states. Additionally, high information density allows for inline quantification and correction of confounding factors such as physiological motion, $T_{2}$, $T_{2}^{*}$, fat fraction, $B_{0}$, and $B_{1}$ with a reduced impact to the overall scan time. However, acquiring data across motion states introduces challenges into the reconstruction process due to the increasing dimensionality of the acquired data. Our thesis with this review is that by applying robust motion characterization and compensation techniques, rich datasets can be acquired that account for multiple confounding factors and accurately map $T_{1}$ among other physiological parameters and confounding factors. For a visual overview of the organization of this review into methods that freeze and compensate for cardiac and respiratory motion, see Table 1.

**Table 1:** Categorization of various approaches to motion correction and motion resolution in T_1_ mapping literature and how they are organized in this review.

Cardiac motion	Respiratory motion	
	Frozen	Compensated
Frozen	2D: Section 3.1	2D: Section 3.3
	3D: Section 4.1	3D: Section 4.2
	Multiparametric: Section 5.1	Multiparametric: Section 5.3
Resolved	2D: Section 3.2	2D: Section 3.4
	3D: Not presented	3D: Section 4.3
	Multiparametric: Section 5.2	Multiparametric: Section 5.4

Methods are categorized by cardiac motion (frozen or compensated), respiratory motion (frozen or compensated) and cardiac motion state (resolved or frozen). In each motion type, methods are further stratified by dimension (2D, 3D) and number of parameters mapped ($T_{1}$, multiparametric). Motion handling techniques impact 2D data different than 3D data due to the handling of through-plane motion, and $T_{1}$ data different from multiparametric data due to the scan time available for parameter quantification.

## 2. Theory

The $T_{1}$ relaxation time is an exponential time constant describing longitudinal magnetization recovery to thermal equilibrium. Specifically, $T_{1}$ is the amount of time required for the longitudinal magnetization to recover to $1-e^{-1}$ of its initial thermal equilibrium value when starting from zero longitudinal magnetization, which can occur immediately after a saturation pulse or a certain time after an inversion pulse. To measure $T_{1}$, multiple samples of the longitudinal spin magnetization $M_{z}$ are acquired after perturbing the initial equilibrium state ($M_{z}^{0}$). The longitudinal magnetization recovers to the initial equilibrium state via the exchange of energy to the surrounding “lattice,” a process known as spin–lattice interaction. The $T_{1}$ value depends on the distribution of energy across the Larmor frequency ω of the protons interacting to produce the longitudinal recovery. Therefore, different field strengths and local disruptions of the Larmor frequency due to metallic implants or paramagnetic contrast agents will change $T_{1}$.

$T_{1}$ mapping is performed by acquiring multiple $T_{1}w$ images at different preparation times to fit for the underlying time constant $T_{1}$ in each voxel. In general, $T_{1}$ relaxation can be modelled as

(1) $$M_{z}(t)=M_{z}^{0}-(M_{z}^{0}-AM_{z}^{0})e^{-t/T_{1}},$$

where $M_{z}(t)$ is the signal intensity of a voxel given the time after the preparation pulse time t, and $M_{z}^{0}$ is the magnitude of the equilibrium state. The constant A describes the ratio between the longitudinal magnetization after a preparation pulse and the equilibrium magnetization. It can be left as a fitting parameter when performing a three-parameter fit, or can be set to A=0 or A=−1 to perform a two-parameter fit when the data is assumed to be initially at thermal equilibrium and the preparation pulse is assumed to be a perfect 90${}^{\circ }$ or 180${}^{\circ }$ pulse, respectively. When used as a fitting parameter, A can capture local differences in the longitudinal magnetization following magnetization preparation that are caused by preparation pulse efficiency or incomplete approach to equilibrium magnetization prior to the preparation pulse.

$T_{1}$ mapping is typically performed voxelwise, and measurements can be further averaged across a region of interest, cardiac segments, or the whole heart. The workflow of $T_{1}$ mapping in the heart has 3 major components: (1) Multicontrast $T_{1}w$ image acquisition, (2) parameter fitting to calculate the $T_{1}$ value based on a theoretical model, and (3) motion compensation and motion correction using the raw $T_{1}w$ images and derived $T_{1}$ maps.

### 2.1. Magnetization preparation for relaxation-based contrast

**Figure 1:**
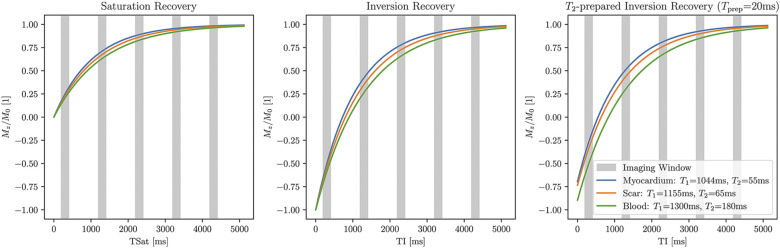
$T_{1}w$ imaging after magnetization preparation. Left: saturation-recovery imaging occurs after a 90${}^{\circ }$ preparation pulse and fits to the curve $M_{z}(t)=M_{0}(1-e^{-t/T_{1}})$. Center: inversion-recovery imaging occurs after a 180${}^{\circ }$ preparation pulse and fits to the curve $M_{z}(t)=M_{0}(1-2e^{-t/T_{1}})$. Right: $T_{2}$-prepared inversion-recovery imaging occurs after an inversion pulse and $T_{2}$-preparation pulse ($T_{\mathrm{prep}}=20$ ms shown here) and fits to the curve $M_{z}(t)=M_{0}(1-(1+e^{-T_{\mathrm{prep}}/T_{2}})e^{-t/T_{1}})$. Signal values from saturation-recovery measurements are restricted to [0, $M_{0}$], whereas signal values from inversion-recovery measurements can take values in [-$M_{0}$, $M_{0}$], leading to increased dynamic range and reduced variability. Representative native $T_{1}$ and $T_{2}$ values at 1.5 Tesla are used for this plot.

Typically, $T_{1}$ contrast is obtained using either inversion-recovery (IR) or saturation-recovery (SR) pulses. For a visual overview of imaging during magnetization recovery after IR or SR pulses, see Figure 1. A combined $T_{2}$-prepared inversion-recovery ($T_{2}$-IR) pulse may also be used when a mixture of $T_{1}$ and $T_{2}$ contrasts is desired, such as for multiparametric mapping.

An IR pulse inverts the initial net longitudinal spin magnetization ($M_{z}$) by 180${}^{\circ }$, resulting in $-M_{z}$ in an ideal situation, with no magnetization in the transverse plane. In practice, residual transverse magnetization and incomplete inversions can occur due to $B_{0}$ and $B_{1}$ inhomogeneities caused by tissue boundaries or implanted metallic devices. To mitigate this, spoiler gradients can be used to dephase any lingering transverse magnetization after the inversion pulse. Spoiler gradients may also be useful pre-inversion and pre-saturation to ensure residual transverse magnetization is not transformed along the longitudinal axis. Imaging readouts are then acquired at different *inversion times* (TIs) after the IR pulse to obtain multicontrast $T_{1}w$ images. Inversion pulses provide increased SNR due to the preservation of the longitudinal magnetization, at the cost of increased scan times due to the recovery time needed for the magnetization to return to the equilibrium value.

The SR pulse consists of a 90${}^{\circ }$ RF pulse to tip the longitudinal magnetization entirely into the transverse plane, where it is then dephased with spoiler gradients. SR differs from IR in that it nulls all signal regardless of the previous magnetization state ($M_{z}$). Therefore, SR pulses minimize contributions of irregular heart rates and heart rhythms when coupled with ECG triggering. Saturation pulses provide improved robustness to heart rate variation and reduction in magnetization recovery time due to the full signal nulling every heartbeat. However, the full signal nulling reduces image SNR, requiring more averages and heartbeats to return high-quality maps.

$T_{2}$-IR pulses can be implemented with a $T_{2}$ preparation module followed by an IR pulse or by replacing the 90${}^{\circ }$ tip-up pulse in the $T_{2}$-preparation module with a 90${}^{\circ }$ tip-down pulse. The magnetization will change from $M_{z}\to -M_{z}\exp(-T_{\mathrm{prep}}/T_{2})$, where $T_{\mathrm{prep}}$ is the $T_{2}$-preparation duration. In this way, images acquired at different TIs and $T_{\mathrm{prep}}$s will have a mixture of $T_{1}$ and $T_{2}$ contrast, making this approach suitable for joint $T_{1}$–$T_{2}$ mapping. $T_{2}$-IR pulses are useful for increased contrast between tissues of varying $T_{2}$, but introduce an obvious confounding factor in the quantitative mapping process. As such, including multiple $T_{\mathrm{prep}}$ values and quantifying both $T_{1}$ and $T_{2}$ may be preferred to quantify $T_{1}$ with the added contrast benefits.

### 2.2. Look–Locker $T_{1}$ mapping

The most accurate approach to generate a $T_{1}$ map is to acquire only one k-space line per acquisition window per TI and subsequently wait until the signal has completely recovered before acquiring the next k-space line. By only acquiring one line at a time and waiting for full signal recovery, perturbation of the $T_{1}$ recovery during the imaging period is minimized, and the signal is allowed to recover close to its thermal equilibrium. As such, this method is preferred for reference mapping in designated $T_{1}$ mapping phantoms, but it leads to impractically long scan times that are not suitable for clinical use.

To make $T_{1}$ mapping more practical, the Look–Locker technique was developed, where a single inversion-recovery pulse is followed by multiple (fully-sampled or undersampled) readouts at each TI (22). Usually, only one TI is acquired during each heartbeat, so timing the center of k-space to the optimal $T_{1}$ contrast for that heartbeat is important. However, the $T_{1}$ relaxation process is perturbed intermittently in a Look–Locker sequence due to the multiple excitation pulses after the IR pulse altering the longitudinal magnetization. This will lead to a different relaxation constant $T_{1}^{*}$, denoting the *apparent* $T_{1}$, and a new steady state $M_{\mathrm{ss}}$ that is distinct from the thermal equilibrium $M_{z}^{0}$. The signal behavior is described as:

(2) $$M_{z}(t)=M_{\mathrm{ss}}-(M_{\mathrm{ss}}-AM_{z}^{0})e^{-t/T_{1}^{*}},$$

and the relationship between apparent $T_{1}$ and actual $T_{1}$ can be derived as (23):

(3) $$T_{1}^{*}={\left(\frac{1}{T_{1}}-\frac{1}{T\;R}\log(\cos(\alpha ))\right)}^{-1},$$

where α is the flip angle of the excitation pulse. This can be further approximated with a correction factor:

(4) $$T_{1}=\left(\frac{M_{\mathrm{ss}}-AM_{z}^{0}}{M_{\mathrm{ss}}}-1\right)T_{1}^{*},$$

which provides a simple way to obtain the actual $T_{1}$ with a three-parameter fit for $T_{1}^{*}$, $M_{\mathrm{ss}}$, and $AM_{z}^{0}$. Although the above derivation assumes a GRE sequence, the correction factor works empirically for bSSFP sequences with low flip angles as well (24).

Inversion-recovery $T_{1}$ mapping sequences with Look–Locker acquisitions can lead to poor $T_{1}$ characterization for long $T_{1}$ values, requiring corrections during the fitting for patient heart rate. Each image for a given TI is acquired in a different cardiac cycle, which can result in misalignment due to superior-inferior motion of the heart linked to respiration. To further improve $T_{1}$ accuracy, corrections due to inter-TI cardiac motion or corrections that disentangle complex interactions between $T_{1}$ and TR, flip angle, RF-inhomogeneity and $T_{2}$ can be used.

### 2.3. Sampling patterns

**Figure 2:**
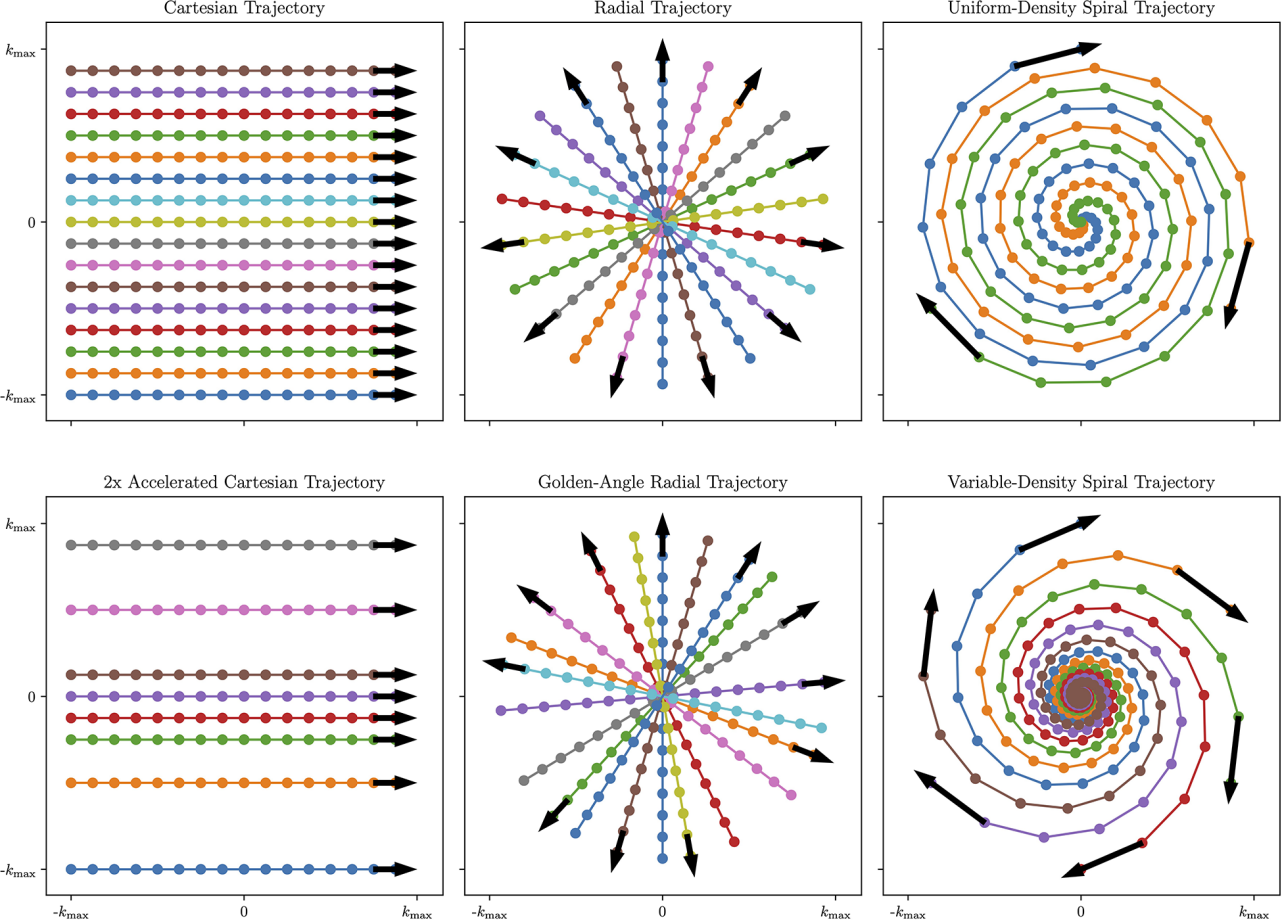
Visual overview of Cartesian and Non-Cartesian k-space sampling trajectories. Left column: Cartesian imaging acquires data line-by-line. This can be accelerated by removing lines from higher-order k-space while preserving lines from lower-order k-space to achieve a variable-density pattern that is optimal for undersampled reconstruction (Bottom Left). Center column: Radial imaging acquires data in spokes traversing the center of k-space. To further increase temporal incoherence and maximize reconstruction flexibility, radial spokes can be rotated by the golden angle $\varphi \approx 111.25^{\circ }$ (Bottom Center). Right column: spiral imaging acquires data in spiral patterns that improve k-space coverage but require time-varying gradients. To further improve motion characterization, variable-density spirals collect more data near the center of k-space and less data in the periphery of k-space (Bottom Right). Figure generated using demonstration materials from the BART toolbox.

The choice of k-space sampling pattern affects the readout duration, sensitivity to motion, sensitivity to $T_{1}$ contrast changes, and total acquisition time among other factors. For further details on the k-space geometry of the proposed patterns, see Figure 2.

#### 2.3.1. Cartesian sampling

Traditional clinical methods acquire the data in a Cartesian or rectilinear trajectory for uniform k-space sampling that leverages a simple fast Fourier transform (FFT)-based reconstruction. Cartesian images tend to be susceptible to motion artifacts, as different motion states acquired in the same image result in coherent ghosting, which may cause the image to be non-diagnostic. However, Cartesian trajectories are more robust to eddy currents and gradient delays than their non-Cartesian counterparts.

#### 2.3.2. Non-Cartesian sampling

Non-Cartesian trajectories may be used to more frequently acquire low-frequency k-space that resolves variations in image contrast and image motion over time in the data. With center-out trajectories such as radial or spiral acquisitions, intra-scan motion tends to manifest as blurring as opposed to ghosting. Additionally, center-out trajectories can easily be reconstructed at a higher field of view (FOV) by oversampling the Cartesian grid during the non-uniform gridding process, which reduces the prevalence of coherent wraparound artifacts. This makes center-out non-Cartesian trajectories potentially useful choices when acquiring $T_{1}$ maps in the presence of cardiac and respiratory motion. In addition, non-Cartesian sequences can be beneficial for undersampled reconstruction techniques such as compressed sensing due to the incoherent nature of aliasing artifacts when compared to Cartesian trajectories. However, non-Cartesian trajectories require computationally intensive reconstructions that may be beyond the power of standard MR scanning workstations.

Radial trajectories are acquired in a series of spokes that, instead of forming a grid, form a disk by passing through the center of k-space with each readout. For fully-sampled radial data, the angle Δθ between neighbouring radial spokes needs to be chosen to meet the Nyquist criteria at a given FOV: $\Delta \theta \leq 1/(\mathrm{FOV}\cdot k_{\max})$. Usually, this requires π/2≈1.57× as many readouts as in a Cartesian acquisition with the same FOV and resolution. However, when performing undersampled reconstruction with radial trajectories, the amount of spokes acquired can be reduced, and uniform k-space coverage across multiple retrospectively selected bins is most important. k-space data acquired in similar cardiac motion states, respiratory motion states, and $T_{1}$ contrast states can be combined into a single bin, defining the data to be used in a given reconstruction, that allows for reduced acceleration factors.

One common choice for flexibly maximizing k-space coverage for undersampled reconstruction across multiple bins in radial imaging is golden-angle rotation (usually referred to as golden-angle radial imaging), where each successive spoke is rotated by the golden angle $\varphi \approx 111.25^{\circ }$ (25). This sampling pattern enjoys the property that each successive radial spoke divides the largest remaining angular space roughly in half, minimizing gaps in sampling space over any given acquisition window (26).

With the oversampled k-space center, radial trajectories can also be used for self-navigation and motion correction (27). Radial imaging can be extended to 3D using either a stack-of-stars readout, where a phase-encode step is used in the $k_{z}$ dimension, or 3D radial “kooshball” sampling, where radial spokes are embedded in a three-dimensional space and identified by two angles (θ,ϕ) on the unit sphere instead of a single angle θ on the unit circle.

Spiral trajectories are an extension of radial trajectories where the radial spokes are twisted from the center of k-space using time-varying gradients so that the maximum distance between each spiral is less than 1/FOV. This improves k-space coverage and sampling density, allowing for highly-accelerated imaging. In addition, variable-density spiral imaging can be used where the center of k-space is more densely sampled, and the periphery of k-space is less densely sampled. This further improves undersampled reconstruction performance by including more low-frequency k-space data that encodes motion and image contrast. Spiral trajectories can be extended to 3D with stack-of-spirals readouts, which incorporate a phase-encode step in the $k_{z}$ dimension; 3D cones readouts, which blend a 2D spiral readout with a 3D radial readout in a highly-efficient manner (28); or infinitely configurable implementations of rotating a single spiral or cone in 3D space.

#### 2.3.3. Single shot sequences

Single shot sequences acquire the entire k-space data after a single preparation pulse and are often used to rapidly acquire 2D slices for $T_{1}$ mapping. To avoid temporal blurring caused by $T_{1}$ relaxation, a short effective acquisition window is required, and parallel imaging is useful to reduce acquisition time for each $T_{1}w$ image. 3D single-shot sequences are generally unfavorable due to the large number of encoding lines required in a small temporal window. Single shot sequences are typically acquired with Cartesian trajectories, but other trajectories such as echo-planar imaging or (variable-density) spirals have been used for specialized applications.

Single-shot sequences have their trade-offs when compared to multi-shot sequences. Notably, single-shot sequences tend to have limited resolution, and longer acquisition windows increase the sensitivity to within-shot motion in addition to changes in $T_{1}$ contrast. However, since data for each TI is acquired in its own shot, inter-shot motion can be dealt with during the mapping analysis as opposed to in the k-space data.

### 2.4. Clinical standard $T_{1}$ mapping sequences

Modified Look–Locker Imaging (MOLLI) is a widely-used cardiac $T_{1}$ mapping sequence (29). Typically, MOLLI uses bSSFP readouts with ECG triggering to minimize cardiac motion and breath-holding to minimize respiratory motion; however, GRE MOLLI has been proposed for certain applications (30). Magnetization transfer effects can lead to a ∼15% underestimation of $T_{1}$ when compared to saturation-recovery estimates in native myocardium. There are different versions of this sequence that modify the number of Look–Locker blocks and signal recovery periods between blocks, but the most used variant is MOLLI 5(3)3, i.e. acquire five TIs in separate heartbeats after an inversion, wait three heartbeats, and acquire three more TIs after another inversion (19). This variant takes 11 heartbeats to acquire and only requires a single breath-hold.

To further reduce scan times, shortened MOLLI (shMOLLI) has been proposed with a 5(1)1(1)1 acquisition scheme that only takes 9 heartbeats (31). shMOLLI is best suited for short-$T_{1}$ mapping, as a recovery period of only 1 heartbeat can lead to errors for long $T_{1}s$. This limitation can be overcome with conditional data analysis, where only signals from the second and/or third TI are used if the $T_{1}$ values are short enough.

An alternative to MOLLI is SASHA, which also uses bSSFP readouts, but SR preparation pulses instead of IR pulses (32). SASHA mapping acquires one unsaturated image followed by one image per heartbeat at multiple saturation times. SASHA does not require recovery heartbeats and is more robust to variations in heart rate. One limitation of SASHA is that the dynamic signal range in SASHA is half of that in IR sequences (Figure 1), which leads to higher $T_{1}$ variability than inversion-recovery methods such as MOLLI (17).

### 2.5. Post-processing and analysis in $T_{1}$ mapping

After acquiring multiple $T_{1}w$ images, least-squares fitting can be used to estimate the underlying $T_{1}$ from voxel-wise signal recovery using the appropriate signal equation, such as Equations 2 and 4 for Look–Locker sequences. Common software functions that could be useful for this task include *lsqnonlin()* in MATLAB, *scipy.optimize.curve_fit()* in Python, and *LsqFit:curve_fit()* in Julia.

However, each sequence requires its own signal equation that may not always be readily computable, as in more sophisticated pseudorandom sequences used in MR Fingerprinting (MRF) (33). A more generalized alternative for parameter estimation is dictionary fitting: given a set of sequence parameters q, simulation via the Bloch equations or the extended phase graph method can be used to generate a function $U_{q}$ that maps tissue parameters $P=(M_{0},T_{1},T_{2},\ldots )$ to the signal evolution $U_{q}(P)$ of a voxel with the input tissue parameters. One can vary the tissue parameters over some domain P to create a dictionary

(5) $$D_{q}=\{(P,U_{q}(P))\;:\;P\in P\}.$$

Note that this dictionary depends on the individual sequence parameters, and often the patient-specific sequence timings (e.g., RR interval), so a new dictionary will often need to be simulated for each sequence and for each patient. To find tissue parameters that correspond with a given fingerprint $u_{0}$, inner product matching can be used to find the closest fingerprint in the dictionary:


(6) $$\hat{P},\hat{U}=\arg\max_{(p,u)\in D_{q}}\langle u,u_{0}\rangle .$$

Other template-matching algorithms that efficiently search the simulated parameter space may be useful to improve computation time (34, 35), but dictionary generation is computationally intensive and requires many simulations, the number of which grows exponentially with the number of tissue parameters. Deep learning-based parameter fitting and dictionary simulation drastically accelerates computation times, which may potentially provide a solution to the computational demands of dictionary fitting (36, 37).

Once $T_{1}$ maps are generated, either from the built-in scanner reconstruction methods or offline post-processing methods, they are usually analyzed with the standardized AHA 16-segment model: six basal segments, six mid-ventricular segments, and four apical segments are determined from three or more 2D slices (38). Global $T_{1}$ can be obtained by averaging over all segments, and segmental $T_{1}$ can be used to localize focal diseases such as cardiac sarcoidosis where pathology is localized within a contained region in the heart.

### 2.6. Motion compensation methods

Conventional methods to freeze motion in clinical $T_{1}$ mapping sequences reduce information density and are not robust to variable heart rhythms and patient inability to comfortably perform repeated breath holds. These tradeoffs can be mitigated by using free-running acquisitions that scan data continuously across cardiac phases with no prospective ECG triggering and free-breathing acquisitions that scan data across respiratory phases without asking the patient to hold their breath. Free-running and free-breathing sequences incur cardiac and respiratory motion artifacts that hinder image quality if not accounted for in acquisition and reconstruction process. In general, cardiac and respiratory motion can be *frozen*, by use of ECG-gating and/or breath-holding, or *compensated* in reconstruction by acquiring data across different motion states. Motion compensation techniques to characterize and handle cardiac and respiratory motion can largely be categorized into *motion-resolved* and *motion-corrected* methods. Motion-resolved sequences present data in multiple motion states to display the physiological motion, whereas motion-corrected sequences present a single dataset that is processed from multiple motion states. This section gives an overview of existing methods in the literature for cardiac MRI motion compensation before we discuss their uses in $T_{1}$ mapping in Sections 3-5.

**Figure 3:**
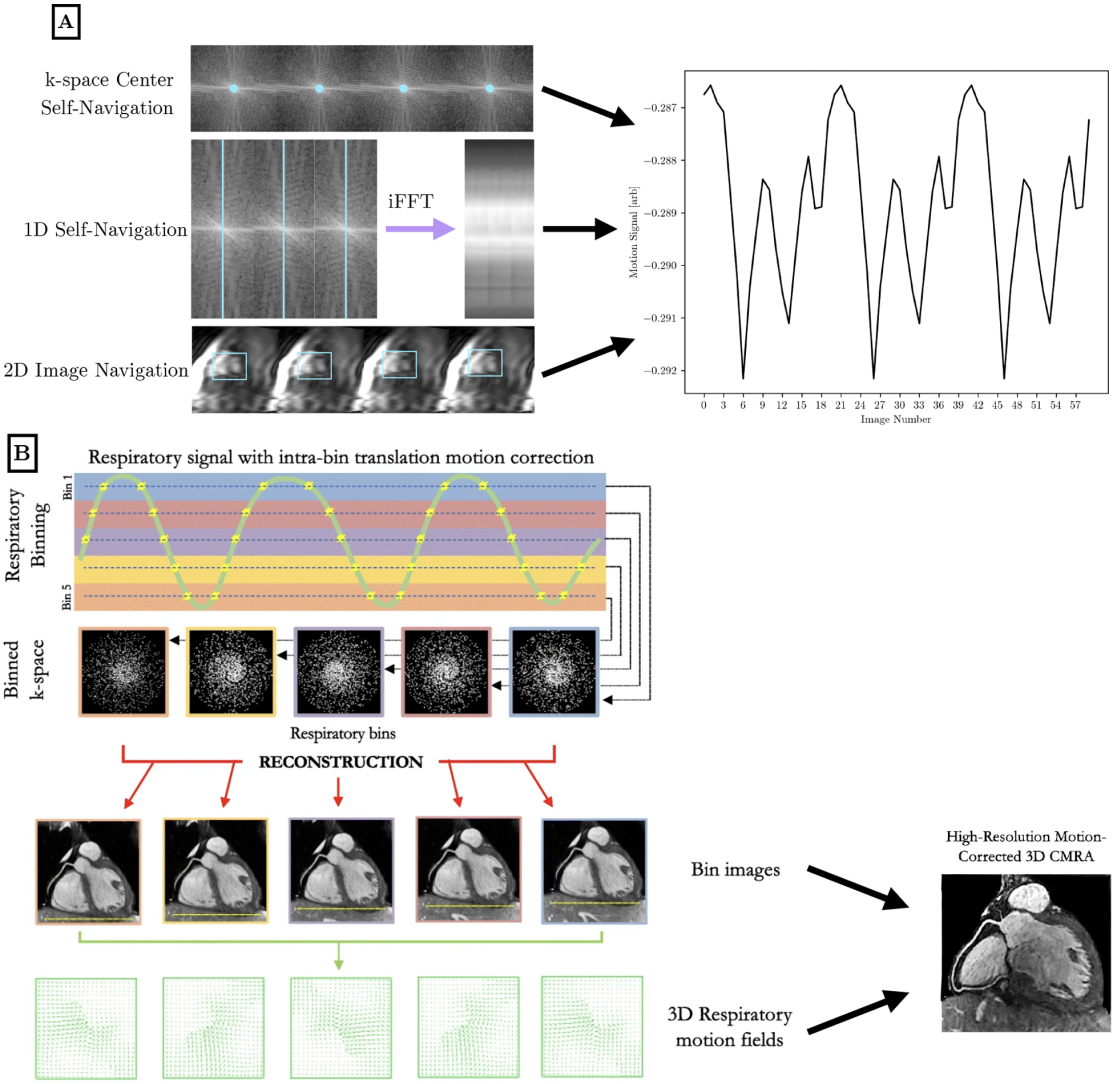
Visual overview of motion characterization (**A**) and motion compensation (**B**) methods in the literature for high-dimensional cardiac MRI. (**A**) Cardiac and respiratory motion proxies can be generated from repeated central k-space data, a repeated 1D spoke, or 2D image navigation images, among other possible methods. The amplitude or phase of the repeated central k-space signal can provide motion information across the entire field of view. A repeated 1D k-space spoke provides a 1D projection that can be used for self-navigation. Low-resolution 2D image navigators are frequently acquired, and the heart is localized using template matching or image registration. Motion parameters are extracted and used to inform future analysis. Optionally, 1D self-navigation and 2D image navigation can be used for rigid-body k-space corrections due to the quantitative motion displacement obtained. (**B**) Respiratory motion proxies can be divided into multiple bins, so that each bin corresponds to data that is acquired at a similar respiratory state. A motion-resolved reconstruction is performed to generate representative images from each bin. Optionally, nonrigid inter-bin registration can generate a single motion-corrected volume from the multiple motion-resolved images. This general workflow is applicable to cardiac motion as well using a cardiac-specific proxy. B is adapted from (21) under the terms of the Creative Comments CC BY License.

To perform motion compensation, surrogate signals of physiological motion must be generated to bin the acquired k-space data into the different motion states. Cardiac motion characterization is often performed with triggering to the QRS peak in the ECG waveform, where the time since the cardiac trigger can be used to bin data into different phases of the cardiac cycle. Respiratory motion characterization can be performed via the respiratory bellows signal that reflects chest expansion—higher values of bellows signal correspond to respiratory states closer to end-inspiration. For a visual reference of motion characterization and motion correction techniques, see Figure 3.

As an alternative to motion characterization using external devices (ECG, bellows), cardiac and respiratory motion can be determined directly from the k-space data, which is referred to as *self-gating*. Center-out trajectories such as 2D radial, 2D spirals, 3D radial, and 3D cones frequently acquire the center of k-space, which corresponds to the summation of all pixels in an image. Protons moving under cardiac and respiratory motion encounter different regions of coil sensitivity that impact the central k-space signal. Monitoring changes in the amplitude or phase of the central k-space data allows for motion characterization without the collection of extra data (39). This can be extended to a 1D navigator using a repeated k-space spoke, which is sometimes oriented in the $k_{z}$ dimension but can also be at an arbitrary angle that crosses the center of k-space. Rapid temporal resolution is needed to capture the relevant motion, so the readout must be repeated on the order of every 50 ms to characterize cardiac motion. In self-gated sequences, cardiac and respiratory motion may be isolated using band-pass filters and principal component analysis, although specialized analysis is needed to isolate motion from $T_{1}$ recovery dynamics.

More recent approaches to motion characterization include image navigators and pilot-tone navigation. Image navigators are an extension of 1D respiratory navigators to include low-resolution 2D or 3D volumes acquired every heartbeat to isolate respiratory motion in a ECG-triggered scan (40, 41). These volumes can be registered together to correct for rigid or nonrigid motion. Pilot tone navigation uses a similar approach to self-gating sequences, where a dedicated readout is played continuously at a frequency outside of the imaging band to acquire signals reflecting changes in motion at a finer temporal resolution than self-gating allows for (42).

Motion-resolved imaging reconstructs multiple 2D/3D volumes that correspond to different motion states. For example, cardiac cine imaging is not a mapping technique, but is a key example of cardiac motion-resolved imaging. Respiratory motion-resolved imaging is often used in free-breathing acquisitions, where multiple binned images are acquired in different portions of the respiratory cycle. Typically, data binned for a given motion state are undersampled representations of the slice or volume; therefore motion-resolved imaging is often performed in conjunction with temporal transform sparsity-based or low-rank reconstruction methods that take advantage of correlations between motion states to improve the reconstruction image quality.

Undersampled data from multiple motion states can be transformed and combined into one motion-corrected 2D/3D volume. Often, motion correction is performed on a collection of motion-resolved images via image registration (43, 44).

In addition to motion correction in image space, translational motion correction can also be applied to k-space data K using the following relation:

(7) $$K^{\prime }=K\exp(2\pi i\vec{k}\cdot d\vec{r}),$$

where $\vec{k}$ denotes the location in k-space, and $d\vec{r}$ denotes the inputted displacement vector. This correction step can be performed at a heartbeat-by-heartbeat level, or more frequently, with self-gated or pilot-tone motion characterization methods. Additionally, iterative optimization can be performed to find the motion parameters that minimize image blur in a region of interest: this is the principle of autofocus motion correction (45). k-space motion correction techniques are inherently global in nature, so locally nonrigid phenomena such as cardiac motion are difficult to approximate by globally affine transformations. Whereas, respiratory motion, despite being only locally rigid, can be effectively corrected by applying global phase shifts across bins to improve image quality within a given region of interest. Additionally, k-space motion correction techniques may not be as accurate as image-space registration between multiple bins, due to the robustness and power of current nonrigid registration methods.

## 3. 2D $T_{1}$ mapping

In this section, we examine methods to account for motion in 2D $T_{1}$ mapping. First, we introduce 2D $T_{1}$ mapping methods that correct for residual respiratory motion in a breath-hold, often due to diaphragmatic shifts in patients who struggle to hold their breath. Next, we introduce 2D $T_{1}$ mapping methods that acquire data across the entire cardiac cycle as opposed to a single cardiac phase. Finally, we introduce fully free-breathing 2D $T_{1}$ mapping techniques and free-breathing techniques that acquire continuously across all cardiac phases. A summary of 2D $T_{1}$ mapping approaches as well as a discussion of the limitations of 2D $T_{1}$ mapping methods is at the end of this section.

### 3.1. Respiratory motion correction within breath-holds

While the primary method to correct for respiratory motion is breath-holds, there can still be residual motion inside a breath hold due to diaphragmatic drift or inability to complete the breath hold. This compromises mapping quality; techniques to correct for this residual motion can reduce the number of re-acquired scans due to non-diagnostic motion artifacts.

**Figure 4:**
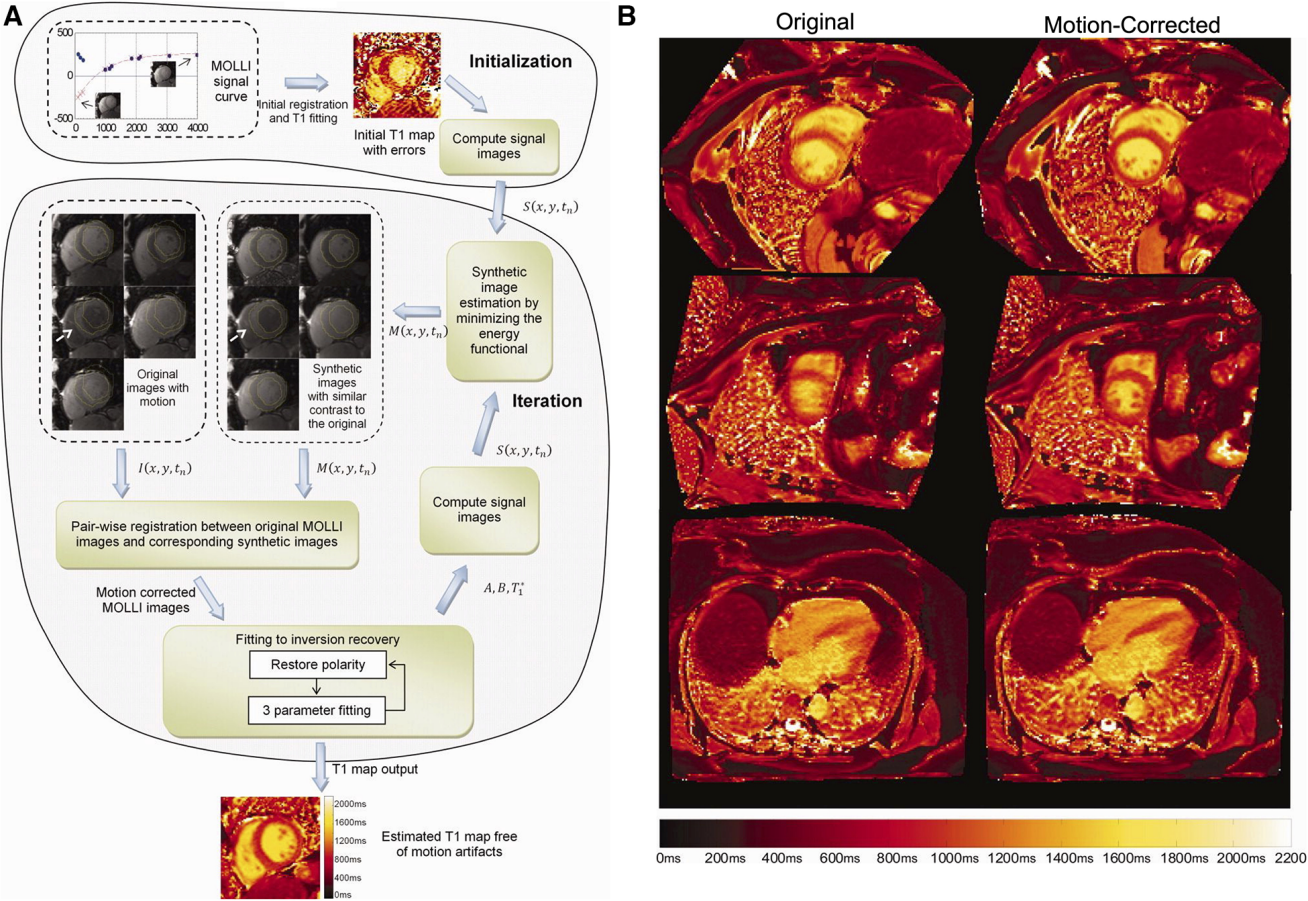
Demonstration of 2D breath-held respiratory motion correction with synthetic image registration. LHS: (**A**) MOLLI data was acquired, and an initial $T_{1}$ map was computed to generate synthetic $T_{1}w$ images in a single motion state. Nonrigid image registration was used to align the acquired MOLLI images with the synthetic images, creating a new $T_{1}$ map with the warped images. This process was repeated iteratively until convergence, usually around three to five iterations. RHS: (**B**) Uncorrected $T_{1}$ maps (left column) compared to motion-corrected $T_{1}$ maps (right column) in short-axis and four-chamber views. Significant respiratory motion was observed despite the scan taking place during a breath-hold. Figure adapted with permission from (46).

Xue et al. proposed a joint motion correction and three-parameter $T_{1}$ mapping approach using synthetic image registration (46). Using a preliminary $T_{1}$ map, synthetic images were generated by solving an energy minimization equation. Polarity corrections at the sample TIs were performed, followed by motion correction between the synthetic images and the acquired MOLLI images using a multi-scale non-rigid fast variational image registration framework. The final pixel-by-pixel $T_{1}$ map was computed from the motion-corrected images using the Nelder-Mead method, with a per-slice reconstruction time of 10 s. For a visual overview of the workflow and sample motion-corrected images, see Figure 4. This work was extended by performing a phase-sensitive image reconstruction to correct the contrast of different inversion images within a MOLLI series (47). Because phase-sensitive IR images have similar contrast across a wide range of TIs, the synthetic image generation step can be removed, and the *T*_1_*w* images can be registered to a common frame. This accelerated the reconstruction time to within five seconds per slice. Roujol et al. proposed an adaptive registration method using rigid and nonrigid corrections to account for residual respiratory motion in a preliminary patient cohort (48). Delso et al. implemented a synthetic image registration approach in a 5(3)3 MOLLI acquisition scheme and assessed the performance using a novel quantitative metric that counted the number of voxels with low T1 fitting residual error (49). Motion correction was performed in an iterative manner with non-rigid registration of the *T*_1_*w* images. In the first iteration, the images were registered to a common reference frame using the median image; in the second and third iterations, the images were registered to synthetically generated images from the first motion state.

Deep learning methods have also been explored for 2D $T_{1}$ mapping: Gonzales et al. used $T_{1}w$ images to train a coarse-to-fine image registration deep learning network (50). Deformation fields were used to correct motion artifacts, and the proposed deep learning method was shown to outperform standard methods with images with severe motion artifacts. Motion correction and mapping took about 30 s per 2D slice. Li and Wu et al. also showed a self-supervised registration method with contrast separation to improve motion characterization in $T_{1}$ mapping (51). The proposed deep learning registration approach was implemented on a GPU and shortened the computation time from 3.7 to 0.5 s. Additionally, an experienced cardiologist evaluated image quality of $T_{1}$ maps and $T_{1}w$ images, finding an increase in image quality and reduction in motion artifacts when compared to standard motion correction methods.

### 3.2. Cardiac motion-informed $T_{1}$ mapping

Conventional cardiac $T_{1}$ mapping techniques like MOLLI only obtain a $T_{1}$ map from a single cardiac phase, but generating $T_{1}$ maps across cardiac phases can acquire comparable $T_{1}$ maps while providing additional physiological information that reduces the need for separate functional cine scans.

Early work to combine $T_{1}$ inversion-recovery contrast and cardiac motion scans simultaneously assessed cardiac function and fibrosis. Connelly et al. used a cardiac-gated, segmented IR-bSSFP sequence to simultaneously produce images at multiple TIs across all phases of the cardiac cycle to assess myocardial viability and wall motion (52). Gupta et al. acquired breath-held IR-cine images with a segmented k-space bSSFP sequence to obtain the optimal TI for LGE MRI (53). Milanesi et al. modified the original IR-cine sequence to allow for both native and post-contrast myocardial $T_{1}$ measurements (54).

Schmidt et al. used a real-time four-second breath-hold IR-cine sequence to jointly evaluate cardiac function and $T_{1}$ recovery (55). The last RR interval where the $T_{1}$ signal has mostly recovered was used to determine displacement fields between cardiac phases, which were propagated to other RR intervals and high-resolution averaged acquisitions to generate a cine series at every TI and for the $T_{1}$ map. Recently, Weingärtner et al. proposed a method to generate cardiac-resolved LGE imaging that made use of multiple $T_{1}w$ images at each cardiac phase to generate auxiliary cardiac-resolved $T_{1}$ maps and $M_{0}$ images (56). These images were used to create synthetic LGE images at each cardiac phase to jointly visualize cardiac function and cardiac fibrosis.

Becker et al. used a golden angle radial sequence to continuously acquire cardiac k-space data with an IR preparation pulse (57, 58). ECG gating was used retrospectively to select specific cardiac phases at multiple TIs. In their first paper, model-based iterative image reconstruction and $T_{1}$ mapping was used to obtain accurate $T_{1}$ maps in a 16-s breath-hold (57). In the second paper, cardiac motion-corrected $T_{1}$ mapping was performed using motion fields which were generated from reconstructed cine data (58). Similarly, Wang et al. used a continuous golden-angle radial acquisition to perform a sparsity-constrained model-based reconstruction that was used to generate a $T_{1}$ map with only 4 s of acquisition (59).

### 3.3. Free-breathing 2D $T_{1}$ mapping

Acquiring multislice $T_{1}$ mapping data requires multiple breath-holds; alternatively, free-breathing acquisitions can improve patient comfort and reduce operator interactions. In this section, we discuss extensions of traditional 2D single-shot mapping techniques to free-breathing acquisitions.

Tsai et al. developed a free-breathing MOLLI sequence by extending the Look–Locker acquisitions to take place over 29 heartbeats (60). Instead of the usual 5(3)3 or 3(3)3(3)5 acquisition patterns, free breathing MOLLI uses a 5(3)5(3)5(3)5 pattern, allowing for redundancy in acquiring multiple images with similar inversion times. Images were averaged to create a mean image, which is used to synthesize $T_{1}w$ images and conduct rigid image registration to correct for in-plane motion. Least-squares fitting is performed to quantify $T_{1}$, and images with high residuals, likely due to through-plane motion, were rejected.

Chow et al. adapted the SASHA sequence for a free-breathing acquisition by incorporating motion correction into a variable-flip angle acquisition (61). In FB-SASHA, a target flip angle of 120${}^{\circ }$ was used with two singular contrasts: no saturation pulse and saturation pulse with a delay of 650 ms. At both saturation times, k-space acquisition used the following pattern: low-frequency k-space acquisition, high-order k-space acquisition, repeated low-frequency k-space acquisition. This pattern was used to create two images at each saturation time: one “primary” image using the first low-frequency acquisition and the high-order acquisition and one “high-contrast” image using the second low-frequency acquisition and the high-order acquisition. The high-contrast images were subtracted from the primary images to create difference images that are used for image registration. Rigid registration was used to reject images with high displacement, so that more data is accepted (around 91%) than a comparable respiratory navigator approach (around 49%). Nonrigid registration between the difference images was then used to correct for in-plane motion before a final $T_{1}$ mapping.

### 3.4. Free-breathing cardiac-resolved 2D $T_{1}$ mapping

Besides correcting or resolving respiratory motion in the free-breathing scan, researchers are also interested in resolving cardiac motion in continuous free-running free-breathing scans, which can maximize the information acquired in a scan while improving patient comfort.

Wang et al. proposed a cardiac motion-resolved model-based reconstruction (59) for free-breathing multi-phase myocardial $T_{1}$ mapping using a free-running inversion-recovery radial GRE sequence (62). Self gating was used for respiratory motion estimation, while ECG monitoring was used for retrospective cardiac motion estimation.

Ludwig et al. proposed a cardiac motion-resolved free-breathing joint cine and $T_{1}$ mapping sequence using pilot-tone motion correction (63). A prior 45-s sagittal calibration scan is used to correlate the pilot tone signal with the head-foot and anterior-posterior motion of the heart that allows for through-plane motion correction in 2D $T_{1}$ mapping.

Guo et al. proposed a free-breathing myocardial exercise stress $T_{1}$ mapping method with a continuous radial GRE acquisition and low-rank plus sparsity reconstruction (64). Self-navigation was performed for extracting respiratory motion, and the mid-diastole phase for every cardiac cycle was retrospectively determined from the recorded ECG signal.

**Figure 5:**
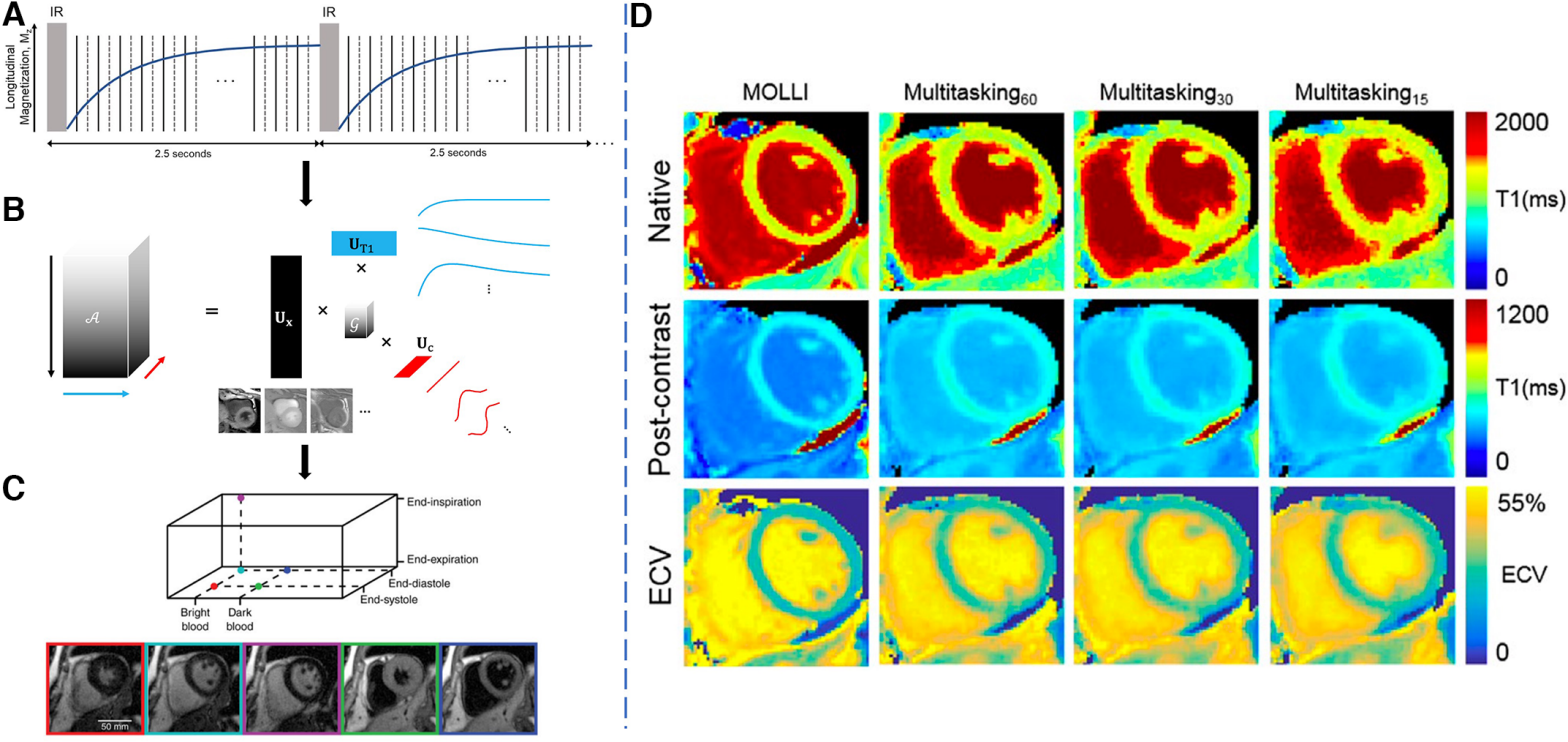
Demonstration of the 2D $T_{1}$ Multitasking framework. LHS: (**A**) Golden-angle radial readouts are acquired continuously after repeated IR pulses. Every other readout, a repeated $0^{\circ }$ radial spoke is collected to resolve temporal dynamics at high temporal resolution. (**b**) A low-rank tensor decomposition is used to represent and reconstruct the multi-dimensional image in a memory-efficient manner favorable for high-dimensional multiparametric imaging. (**C**) Reconstructed images in different cardiac phases, respiratory phases and inversion times. RHS: (**D**) Sample results for native and post-contrast $T_{1}$ maps as well as the ECV maps from MOLLI, 60-s, 30-s and 15-s $T_{1}$ Multitasking. $T_{1}$ mapping quality does not change much moving from 60 s of data to 30 s of data, but reduces slightly from 30 to 15 s, particularly in the post-contrast setting. This causes ECV map quality to decrease with shorter scan times, particularly when moving from 30 to 15 s. Figure adapted with permission from (65, 66).

Christodoulou et al. proposed the MR Multitasking framework that used a self-gated free-breathing continuous IR-GRE radial acquisition and low-rank tensor reconstruction framework to resolve both cardiac and respiratory motion to obtain motion-resolved $T_{1}$ maps (65). Shaw et al. adapted the Multitasking framework to produce pre-contrast and post-contrast cardiac $T_{1}$ mapping with two continuous free-breathing, ECG-free scans (66) that allow for ECV analysis. For a visual overview of the workflow and to see sample multitasking images, see Figure 5. Chen et al. applied deep learning non-Cartesian reconstruction methods to MR Multitasking to accelerate the reconstruction time for improved clinical applicability (67, 68).

### 3.5. Summary of 2D mapping methods

2D $T_{1}$ mapping is fundamentally limited by through-plane motion and slice misalignment, but requires less data than a whole 3D volume. Motion freezing techniques such as ECG-gating and breath-holds simplify $T_{1}$ mapping post-processing, but leave images susceptible to artifacts in the presence of sub-optimal gating or incomplete breath-holds. Post-processing complexity increases when intentionally acquiring in the presence of cardiac or respiratory motion, but the associated use of motion-compensation methods, if sufficiently robust, can lead to more predictable and reproducible results as well as improved patient comfort.

We can collect the most information per unit acquisition time using free-running $T_{1}$ methods that continuously acquire data without regard to cardiac phase and respiratory phase. However, these scans require long total scan time to fully populate the different motion states (approximately 20 cardiac phases × 5 respiratory phases = 100 motion states). To minimize total scan time to acquire multiple 2D slices, free-breathing respiratory motion-corrected cardiac-gated methods may be most beneficial at present. Acquiring data across respiratory phases allows for improved patient comfort and shorter scan times, and exploiting correlations across respiratory bins can improve compressed sensing reconstruction quality. Further, contributions due to cardiac motion are reduced in the imaging and reconstruction.

## 4. 3D $T_{1}$ mapping

Clinical $T_{1}$ maps are currently acquired slice-by-slice, with four-chamber, three-chamber, two-chamber, and multiple short-axis views collected for visualization of the whole heart. However, slices may be misregistered due to variability in the breath-holds or motion states, and slice gaps due to this acquisition scheme may cause focal lesions to go undetected. More slices can be prescribed in the exam, but this will increase the number of breath-holds needed for the patient. One option to accelerate scan times is to acquire multiple 2D slices in a single acquisition using simultaneous multislice excitation and reconstruction techniques (69). An alternative would be to acquire $T_{1}$ maps for a 3D volume in a single scan, reducing errors due to registration and through-plane motion. However, scan times for 3D sequences are necessarily longer than 2D sequences, making high-resolution breath-held 3D acquisitions challenging. As such, 3D free-breathing sequences are often used to collect high-resolution data over multiple respiratory cycles.

### 4.1. 3D $T_{1}$ mapping in a single breath-hold

Performing whole-heart $T_{1}$ mapping at clinical resolutions during a breath-hold requires many optimizations due to the increased amount of data needed. To accomplish this, Warntjes et al. proposed a 3D breath-held $T_{1}$ quantification method using a 3D GRE echo planar imaging readout (70). Whole-heart $T_{1}$ maps with a nominal resolution of 1.5×1.6×5 mm${}^{3}$ were acquired in a 24-s breath-hold. This method is designed to measure short $T_{1}$ values after gadolinium contrast administration, which requires less waiting time for magnetization recovery.

Benatou et al. utilized a multiband excitation pulse to acquire three 2D bSSFP-MOLLI $T_{1}$ maps in a single 11-heartbeat breath-hold (69). Weingärtner et al. used a multiband excitation pulse to acquire three 2D $T_{1}$ maps using a combination of SR and IR pulses in a single 15-heartbeat breath-hold (71). $T_{1}$ values for both methods were shown to be largely consistent with reference single-slice acquisitions, with the combined SR and IR method having improved accuracy. These acquisition strategies allow for a 16-segment AHA analysis from a single breath-hold acquisition.

Hufnagel et al. proposed a method to produce a 3D super-resolution $T_{1}$ map from repeated 2D multislice breath-hold acquisitions (72). A 2D golden-angle radial readout scheme was used with IR magnetization preparation for six slices within a single breath-hold, and the entire acquisition was repeated over eight breath-holds. Motion correction was performed across breath-holds, and a model-based super-resolution reconstruction algorithm was used to generate volumetric $T_{1}$ maps with high in-plane and through-plane resolution. This approach improves the visualization of smaller structures, even with an acquired through-plane resolution of 6–8 mm.

### 4.2. Free-breathing 3D $T_{1}$ mapping

Weingärtner et al. proposed the slice-interleaved $T_{1}$ mapping sequence (STONE) to produce free-breathing 5-slice $T_{1}$ mapping in 2 min at 1.5 Tesla (73). The readout strategy uses a familiar Look–Locker method, but interleaves multiple slices to obtain 2D multi-slice k-space data. Guo et al. improved the volumetric coverage and respiratory motion correction of the STONE sequence to enable 10-slice $T_{1}$ mapping in 2 min at 3 Tesla (74). In-plane motion was retrospectively corrected using image registration, and through-plane motion was prospectively corrected by shifting the plane of imaging according to the respiratory navigator signal. Zhu et al. extended the STONE framework by incorporating dictionary learning that includes $T_{1}$- and $T_{2}$-dependent Bloch simulations. Motion correction used two steps: a linear phase shift in k-space was used to correct for translational motion, and nonrigid optical flow registration was used to correct residual motion between synthetic generated images and acquired $T_{1}w$ images (75).

**Figure 6:**
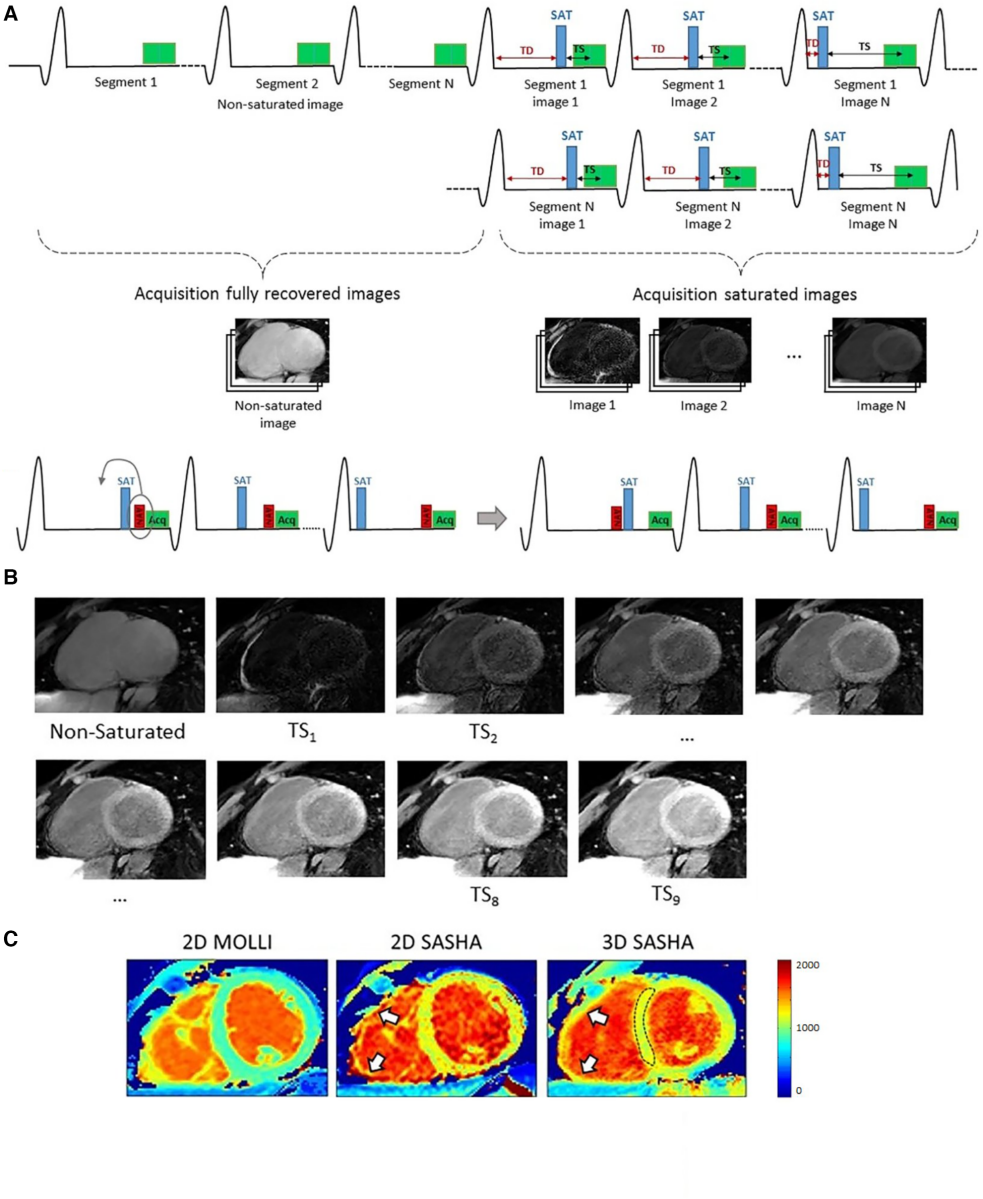
Demonstration of gated 3D SASHA $T_{1}$ mapping. (**A**) 3D SASHA acquisition scheme: A 1D diaphragmatic navigator for respiratory motion compensation was placed before or after the saturation pulse depending on the saturation time. (**B**) Within a nominal scan duration of 4 min 14 sec, nine images were acquired at different saturation times for $T_{1}$ fitting. (**C**) 3D SASHA mapping offers higher resolution compared to 2D techniques; in particular, 3D SASHA can better delineate the right ventricle free wall (white arrows) than 2D SASHA, potentially due to the slightly thinner through-slice resolution of 8 mm instead of 10 mm. Figure adapted from (76) under the terms of the Creative Commons CC BY License.

Nordio et al. extended the 2D SASHA technique to generate 3D high-resolution $T_{1}$ maps using SR $T_{1}w$ imaging and 1D-navigator-based respiratory motion compensation (76). A segmented 3D k-space bSSFP acquisition was used to achieve a resolution of 1.4×1.4×8 mm${}^{3}$ over a duration of 12.0±0.1 min with a respiratory gating efficiency of 32% and a $T_{1}$ accuracy comparable to 2D SASHA. For sample images, see Figure 6. The same group continued to work on improving the precision of 3D SASHA mapping with a 3D Beltrami regularization-based denoising technique and obtained comparable precision to 2D MOLLI *T*_1_ maps (77).

Guo and Chen et al. developed a 3D volumetric $T_{1}$ mapping method at 3 Tesla based on acquiring three SR images. A highly-anisotropic image resolution of 1.5×1.5×16 mm${}^{3}$ was used to cover the heart with six slices in the z direction. Respiratory gating was used, with an average respiratory gating efficiency of 39±11%, for a total scan time of 6.0±1.1 min (78). Recently, this method was improved to include a variable flip angle whole-heart acquisition that improved the through-plane resolution from 16 to 8 mm (79).

Han et al. used a non-Cartesian stack-of-stars trajectory to obtain free-breathing $T_{1}$ maps and $B_{1}^{+}$ maps with a 1.9×1.9×4.5 mm${}^{3}$ resolution in an average scan time of 14.2 min (80). A combination of training data with limited k-space coverage and a high sampling rate and imaging data with full k-space coverage sparsely sampled across respiratory bins was used to track motion and collect imaging data. Data was sorted into eight respiratory bins using a 1D respiratory navigator, and end-exhalation data was used for analysis.

### 4.3. Cardiac-resolved 3D free-breathing $T_{1}$ mapping

Free-running sequences further improve information density by leveraging correlations cardiac motion states and have been shown to generate high-quality high-resolution 3D $T_{1}$ maps. Due to the increased complexity of acquiring 3D data in multiple $T_{1}w$ contrasts at all cardiac phases, these acquisitions are performed using free-breathing techniques that admit longer scan times and leverage robust respiratory motion compensation methods.

Qi et al. developed a free-breathing, free-running 3D myocardial $T_{1}$ mapping with 1.5 mm isotropic spatial resolution (81). This sequence consisted of an inversion-recovery preparation pulse and continuous 3D golden angle radial data acquisition. k-space data was binned into different respiratory states using respiratory motion information extracted from the k-space center of all radial spokes. Bin-to-bin 3D translational respiratory motion was estimated and corrected in k-space using the output of rigid registration. Data was separated into multiple cardiac phases using the simultaneously acquired ECG signal, and $T_{1}$ maps at different cardiac phases were generated with isotropic spatial resolution using dictionary-based low-rank inversion and patch-based reconstruction, with a precision of septal $T_{1}$ similar to MOLLI.

Di Sopra et al. extended the free-running framework for fully self-gated cardiac and respiratory motion-resolved 5D imaging of the heart to add $T_{1}$ recovery contrast as an extra dimension (82). With a single uninterrupted 3D radial GRE acquisition including periodically applied inversion pulses, cardiac-resolved and respiratory-resolved $T_{1}$ maps were achieved at an isotropic spatial resolution of 1.96 mm${}^{3}$ in a 17-min scan. Phantom results showed good agreement with reference sequences for $T_{1}s$ in the 100–1800 ms range, and a preliminary in-vivo study at 3 Tesla also demonstrated comparable myocardial $T_{1}$ values to MOLLI results.

### 4.4. Summary of 3D mapping methods

3D $T_{1}$ mapping is fundamentally limited by the trade-off between choice of through-plane resolution, SNR, and acquisition time within and beyond breath-holds. Isotropic, high-resolution imaging provides the most clinical utility due to the ability to arbitrarily reformat images, eliminating the need to acquire many 2D oblique-angle scans. The most information-dense sequences are 3D cardiac-resolved $T_{1}$ mapping scans, however the $T_{1}$ mapping quality can be reduced due to the increased number of cardiac phases reconstructed. Unlike the 2D imaging case, scan times are similar between cardiac-gated and cardiac-resolved acquisitions, but sufficient data needs to be acquired for multiple inversion times in all cardiac phases, which can be challenging to implement in clinically feasible scan times.

## 5. Multiparametric $T_{1}$ mapping

Multiparametric sequences allow for integrated measurements of confounding factors to $T_{1}$ mapping, such as fat fraction, $B_{0}$ homogeneity, and $B_{1}$ homogeneity; these factors can be resolved into their own separate dimensions, leaving improved $T_{1}$ map quality. Incorporating multiple parameters into the simulation, acquisition, and analysis of parametric mapping techniques allows for more accurate measurements, at the cost of increased computational complexity. In this section, we present motion-informed multiparametric sequences that extend the clinical utility of $T_{1}$ mapping sequences while isolating the confounding factors to $T_{1}$ maps.

### 5.1. Multiparametric mapping within a breath-hold

Early work to combine $T_{1}$ and $T_{2}$ contrast is from Kellman et al., where phase-sensitive LGE imaging is combined with $T_{2}$-weighted imaging to isolate endocardial infarct from blood-pool signal (83). Segmented GRE readouts are used, which alternate between $T_{1}w$ images, proton-density weighted images, $T_{2}w$ images, and a recovery heartbeat. To extend this idea to quantitative imaging, Akçakaya et al. combine SR and T2prep pulses, varying the saturation time and $T_{2}$ preparation time to generate co-registered $T_{1}$ and $T_{2}$ maps for a single slice in a 13-heartbeat breath-hold (84). Maps generated with this strategy were comparable with standard 2D SASHA and T2prep-bSSFP mapping. Santini et al. used real-time IR-SSFP imaging to fit for the steady-state $M_{ss}$ and the effective $T_{1}^{*}$, which is used to fit for $T_{1}$ and $T_{2}$ (85). Recently, Kellman et al. perform joint post-contrast $T_{1}$ and $T_{2}$ mapping in a single 45-heartbeat free-breathing multiparametric SASHA scan (86). These $T_{1}$ and $T_{2}$ maps were used to simulate bright-blood and dark-blood phase-sensitive LGE images.

Another method to acquire quantitative multiparametric maps has been adapting the MR fingerprinting technique originally proposed by Ma et al. for cardiac applications (33, 87). In particular, ECG-gating and breath-holds are used to handle cardiac and respiratory motion. To improve the slice coverage and reduce the number of breath-holds, Hamilton et al. propose a simultaneous multi-slice cardiac MRF sequence with a multi-band factor of 3 and a low-rank reconstruction to improve image quality (88). To correct for intra-diastolic cardiac motion in longer acquisition windows for cardiac MRF, Cruz et al. proposed a framework for low-rank motion corrected (LRMC) MRF (89). Respiratory bellows and ECG signal were used as surrogates for binning the data into different motion states, and images reconstructed for each bin were used for motion estimation. The motion-corrected images were reconstructed by combining the bin-to-bin motion estimation and the low-dimensional Bloch subspace estimated in the MRF acquisition. This technique generates 2D myocardial $T_{1}$ and $T_{2}$ maps, but could also be used for 3D myocardial and liver mapping.

3D co-registered $T_{1}$ and $T_{2}$ maps across the whole heart can be acquired in a single 15-heartbeat breath-hold at a resolution of 2×2×12 mm${}^{3}$ (90) using the 3D QALAS technique. A combination of IR and T2prep modules are used to encode both $T_{1}$ and $T_{2}$ contrast, and simulation-based analysis was used to determine $T_{1}$ and $T_{2}$ from the reconstructed images.

### 5.2. Cardiac motion-informed multiparametric mapping

In the TOPAZ sequence, Weingärtner et al. acquired breath-held continuous IR low-flip angle gradient-echo cine data to simultaneously fit for $T_{1}$ and a $B_{1}^{+}$ surrogate β that relates to the IR pulse efficiency (91). Agreement with reference IR-spin echo measurements was improved by including the $B_{1}^{+}$ correction into the parameter fitting.

**Figure 7:**
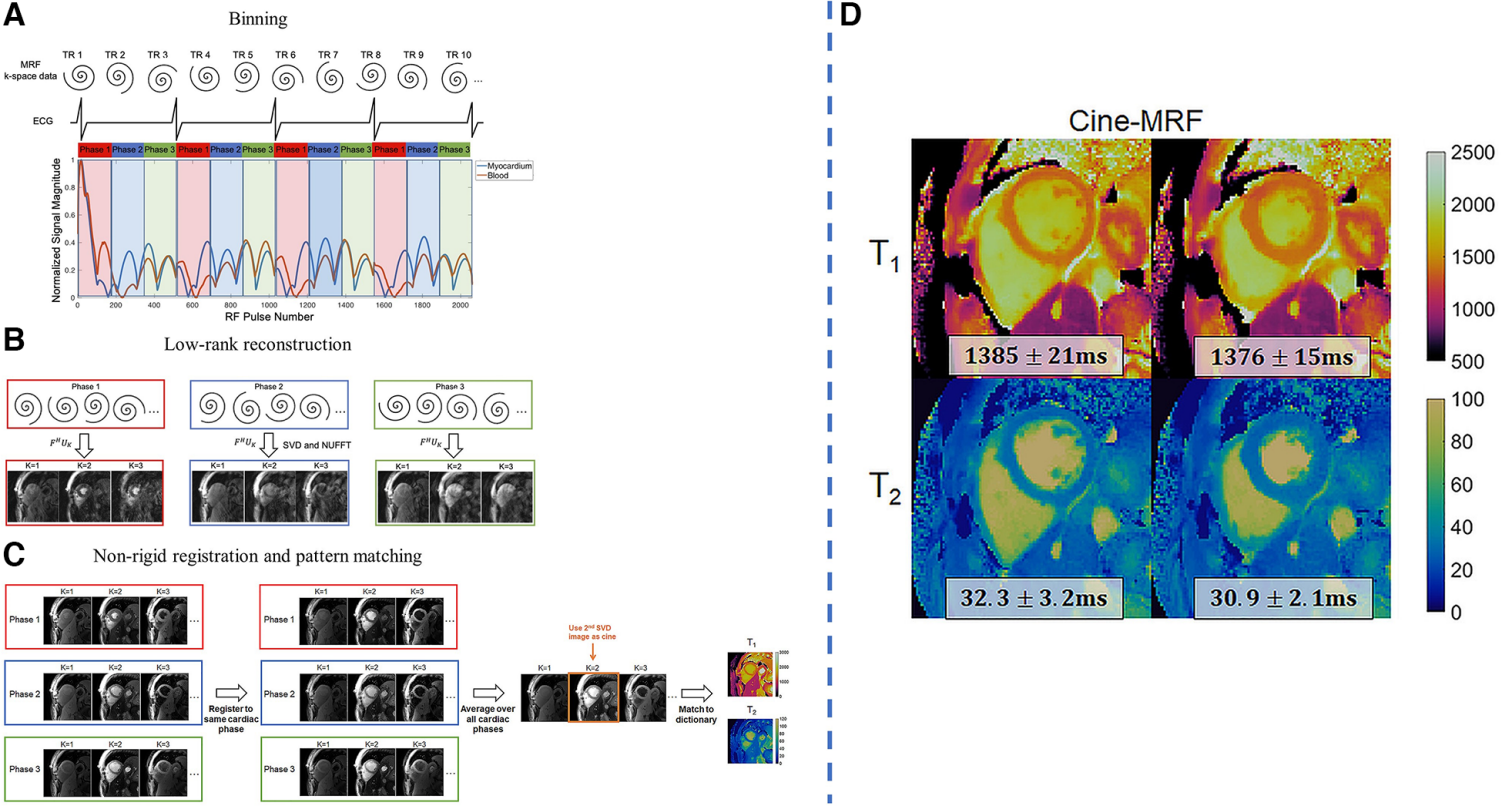
Demonstration of joint 2D free-running $T_{1}$, $T_{2}$ mapping and cine imaging. LHS: (**A**) The collected k-space data is retrospectively binned into different cardiac phases using the ECG signal. Each RR interval is divided into 24 equally-spaced bins, although only three are shown for clarity. (**B**) Reconstruction is performed using a low-rank subspace-constrained reconstruction. (**C**) Subspace images are registered to a target phase with nonrigid deformation fields. The images are averaged and matched to the dictionary to generate mapping results. After registration, the 2nd singular image is used for functional cine analysis. RHS: (**D**) sample results of diastolic and systolic parametric maps. Figure adapted from (93) with permission.

Jaubert et al. developed a continuous, free-running MRF technique using golden-angle radial sampling to jointly acquire cardiac-resolved $T_{1}$ maps, $T_{2}$ maps, and $M_{0}$ cine images at a resolution of 2×2×10 mm${}^{3}$ (92). Due to the long scan time of 29.4 s, the acquisition was performed after hyperventilation to improve breath-hold consistency in volunteers. Data was retrospectively assigned into different cardiac phases with the ECG signal, and reconstruction for each cardiac motion state was performed by combining physics-informed simulated temporal basis functions with patch-based regularization. Similarly, Hamilton et al. developed a free-running MRF technique using spiral k-space readouts for myocardial $T_{1}$ mapping, $T_{2}$ mapping, and cine imaging at a resolution of 1.6×1.6×8 mm${}^{3}$ within a single 16-heartbeat breath-hold (93). Similar subspace constraints were used, together with wavelet regularization along the spatial dimension and finite differences regularization along the cardiac motion dimension. The reconstructed images were nonrigidly registered to the same cardiac phase and were averaged before fitting for better SNR. An overview of the workflow and some sample results is shown in Figure 7.

### 5.3. Free-breathing multiparametric mapping

Guo and Cai et al. extended the STONE framework to include $T_{2}$2-IR preparation, allowing for multislice joint $T_{1}$ and $T_{2}$ mapping with slice tracking and nonrigid motion correction (94). Similarly, Guo and Chen et al. used respiratory navigation to acquire 3D whole-heart $T_{1}$ and $T_{2}$ maps at a resolution of 1.5×1.5×16 mm${}^{3}$ during a 7.9±1.4 min free-breathing scan (95). Five $T_{1}$–$T_{2}w$ contrasts were acquired with saturation pulses, T2prep pulses, both of these pulses, or neither of these pulses, and followed by a six-heartbeat recovery period.

Cruz et al. developed a free-breathing cardiac MRF technique using a variable-density stack-of-spirals readout that enabled 3D voxelwise parametric mapping using traditional cardiac MRF algorithms (96). Signal from the respiratory bellows was used as a surrogate for respiratory motion in an autofocus motion-corrected reconstruction. This approach allowed for whole-left ventricle joint $T_{1}$ and $T_{2}$ mapping at a 2×2×8 mm${}^{3}$ resolution in a 6.9±1.1 min free-breathing scan. Singular images were generated with a joint low-rank inversion and high-dimensional patch-based denoising reconstruction, while parameter maps were generated using dictionary matching from the singular images.

**Figure 8:**
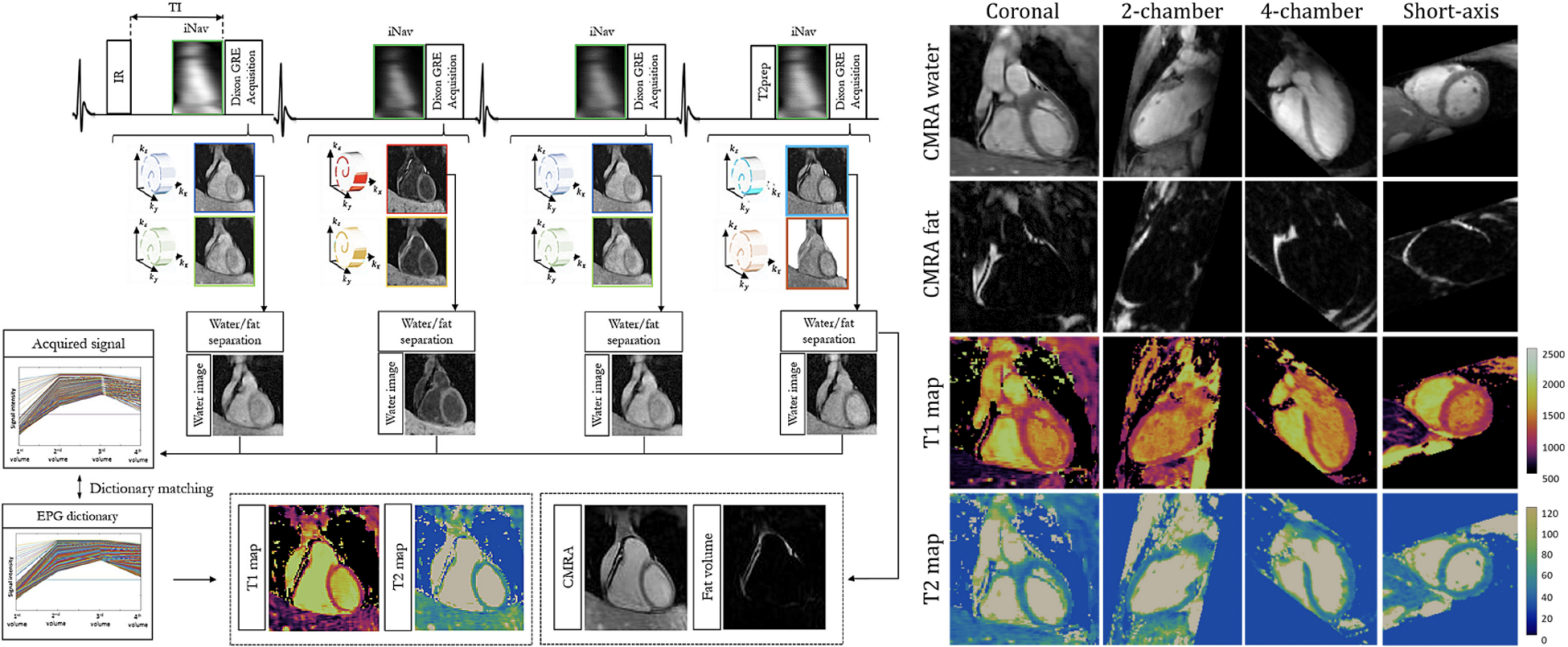
Demonstration of 3D isotropic joint $T_{1}$, $T_{2}$ mapping, coronary MR angiography water/fat imaging. LHS: four singular images are generated using different preparation schemes. A multi-echo acquisition is used to disentangle signal from water and fat. RHS: demonstration of arbitrary multiplanar reformatting to obtain multiple clinically relevant imaging planes with a single isotropic acquisition. Figure adapted from (97) under the terms of the Creative Commons CC BY License.

Milotta et al. used a free-breathing dual-echo variable-density spiral profile order Cartesian sampling pattern to generate eight synthetic images—four $T_{2}$-IR contrasts, each with two echoes—for 2 mm isotropic joint $T_{1}$ and $T_{2}$ mapping, water/fat imaging, and coronary MR angiography (97). Two echoes were used to separate water and fat using the Dixon method, and the four water images with different magnetization preparations were analyzed using dictionary matching to return parametric maps. Fat images and the corresponding water image volumes were used to generate fat fraction maps. 2D image navigators were used for motion correction, allowing for a total scan time of 9±1.5 min at an acceleration factor of R∼4. For a visual overview of this pipeline and sample images, see Figure 8.

### 5.4. Cardiac-resolved free-breathing multiparametric mapping

Zhou et al. used dual-flip angle excitations with a free-breathing continuous spiral IR-GRE pulse sequence to obtain respiratory motion-corrected cine images and $B_{1}^{+}$ slice-profile-corrected $T_{1}$ maps (98). Alternatively, continuous free-breathing IR-GRE data with a single flip angle can be used to generate a Bloch-Siegert shifted $B_{1}^{+}$ map and corrected $T_{1}$ map (99).

To extend 2D cardiac- and respiratory-resolved parametric mapping towards multiparametric acquisition, Christodoulou et al. combined IR and T2prep pulses in a single acquisition for joint $T_{1}$ and $T_{2}$ mapping using the MR multitasking framework (65). Cao et al. further extended the multitasking framework for simultaneous ECG-free $T_{1}$, $T_{2}$, $T_{2}^{*}$, and fat fraction mapping (100). A variable TR scheme was proposed where a single-echo, short TR readout was interleaved with multi-echo readouts that allowed for reduced scan times while maintaining parametric accuracy. Serry et al. acquired dual-flip angle cardiac- and respiratory-resolved multitasking data for $B_{1}^{+}$-corrected $T_{1}$ maps (101). The sequence improved accuracy and repeatability of the $T_{1}$ maps over the previous multitasking method.

To increase the scan coverage in MR multitasking while maintaining a short scan time, Mao et al. modified the 2D multitasking sequence to include a simultaneous multislice excitation pulse (102). This allows for cardiac- and respiratory-resolved $T_{1}$ and $T_{2}$ maps for three short-axis slices in a predictable three-minute scan time. To expand MR multitasking to whole-left ventricle coverage, Mao et al. developed an approach using continuous 3D stack-of-stars readouts with interleaved training and imaging data (103). Cardiac- and respiratory-resolved $T_{1}$, $T_{2}$, and $B_{1}^{+}$ maps were generated at a 1.4×1.4×8 mm${}^{3}$ resolution in a predictable 9.2-min scan time. To improve image quality and SNR, a joint pre- and post-contrast multitasking sequence acquired comprehensive imaging data in a single “push-button” acquisition (104). Here, a $T_{1}$–$T_{2}$ pre-contrast multitasking acquisition was performed for 9.5 min, and a $T_{1}$-only post-contrast multitasking acquisition was performed immediately after for 10.5 min. The evolution of $T_{1}$ contrast after Gd injection was tracked, and the data from 10 min post-injection was used for LGE and ECV analysis. Jointly reconstructing pre-contrast and post-contrast data using shared spatial basis functions allows for improved image quality.

**Figure 9:**
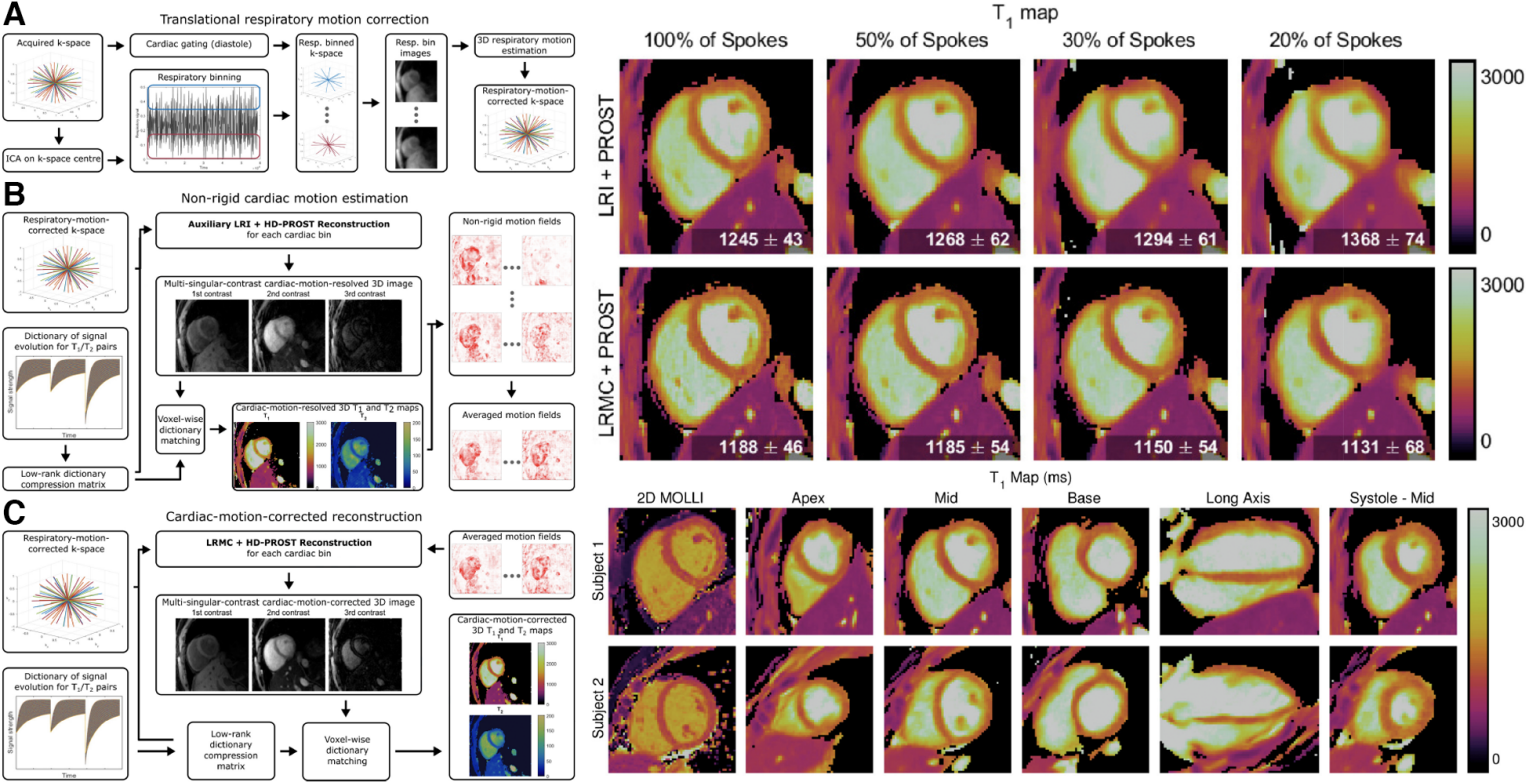
Demonstration of 3D isotropic joint $T_{1}$, $T_{2}$ mapping and cine imaging. LHS: Continuous 3D golden angle radial imaging is acquired with IR and T2prep pulses. Translational respiratory motion correction is performed, followed by nonrigid cardiac motion compensation. A final cardiac motion-corrected reconstruction is performed, and dictionary matching is used to determine $T_{1}$ and $T_{2}$ from the signal intensity of the singular images. The second singular image is used for functional cine analysis. RHS: sample $T_{1}$ maps for a variety of acceleration factors, spatial locations, orientations, and cardiac phases. Note the tradeoff between imaging time and reconstruction quality: as scan time is reduced, the sharpness of the $T_{1}$ maps is also reduced. By increasing the scan time, a higher effective resolution is obtained from the increased amount of higher-order k-space data. Figure adapted from (106) under the terms of the Creative Commons CC BY License.

Qi et al. interleaved IR and T2prep pulses to their prior 3D free-breathing free-running golden-angle radial readout to acquire 2 mm isotropic joint $T_{1}$ maps, $T_{2}$ maps, and cine images in a single 11-min scan (105). Self-gating was used to characterize respiratory motion, which was again corrected in k-space after an image registration step using low-resolution respiratory-binned images. Data was then retrospectively binned into cardiac phases using the ECG signal, and dictionary matching was used for parametric mapping. Phair et al. added nonrigid cardiac motion correction into the reconstruction pipeline, which improved the image sharpness and allowed for higher acceleration factors and shorter scan times (106). Notably, the improved reconstruction allowed for comparable image quality between 11 min of data with prior reconstruction methods and 3.3 min of data with novel reconstruction methods. Additionally, when comparing the prior and novel reconstruction methods with the same 11 min of data, sharper and more detailed images were generated with the novel method. For a visual overview of the workflow and changes in $T_{1}$ mapping quality, see Figure 9.

### 5.5. Summary of multiparametric mapping

2D multiparametric sequences are fundamentally limited by the extended scan time needed to map multiple parameters across multiple slices for clinical scan coverage. To maximize information density, free-breathing free-running multiparametric imaging allows for comprehensive data collection and improved patient comfort due to the removal of breath-holds. However, to keep scan times reasonable for acquiring multiple slices or full left ventricle coverage, an acquisition time of less than one minute per slice is desired.

Similar to 3D single-parametric $T_{1}$ mapping, 3D multiparametric imaging is limited by the choice of acquired resolution and total scan time. Acquiring across cardiac phases is one way to increase the amount of data acquired per unit time, but sufficient dat is needed at each cardiac (and respiratory) phase to generate multiple singular-contrast images and accurate parameter maps. Decomposing spatial dynamics from temporal dynamics may be a viable strategy to reduce the amount of data needed in each bin.

## 6. Discussion

We have presented a review of the imaging physics behind $T_{1}$ measurements and novel methods to measure $T_{1}$ and its confounding factors in the presence of cardiac and respiratory motion. A variety of techniques are employed (a) to improve the ability of encoding 3D k-space across cardiac and respiratory motion states; (b) to correct for or resolve cardiac and respiratory motion; and (c) to reduce errors in voxel-wise $T_{1}$ estimation, including simultaneous acquisition of and correction for potential confounding factors. The ultimate goal is accurate, precise, and reproducible clinically useful $T_{1}$ measurements.

### 6.1. Robust k-space sampling patterns

Many $T_{1}$ mapping sequences use 2D Cartesian single-shot readouts that allow for simpler reconstruction and analysis. However, highly undersampled, motion-compensated acquisition and reconstruction methods benefit from sampling patterns that enable sparsity-based and low-rank reconstruction techniques due to the associated spatial and temporal artifact incoherence. Golden-angle radial readouts densely sample the center of k-space, but are limited in peripheral k-space coverage that may degrade $T_{1}$ mapping quality for highly accelerated acquisitions. 2D spiral acquisitions acquire more data in the periphery of k-space, and variable-density spiral acquisitions improve on the coverage of low-frequency k-space.

Variable-density Cartesian sampling patterns have been proposed to densely sample the center of k-space while incoherently sampling the periphery of k-space, enabling highly-accelerated 3D Cartesian imaging (107–109). Alternatively, 3D stack-of-stars sampling has been proposed to improve retrospective binning flexibility. Stack-of-stars sampling density is usually uniform in the $k_{z}$ dimension, but suffers from the same limited in-plane peripheral sampling density as 2D radial sampling. Stack-of-spirals sampling patterns can improve the in-plane sampling density uniformity, and variable-density acquisitions along the $k_{z}$ dimension can be used to further accelerate scan times. 3D radial sampling provides excellent retrospective binning flexibility, but $T_{1}$ mapping quality is severely degraded in highly accelerated scans due to the limited sampling in the periphery of k-space. 3D cones sampling has been proposed to combine aspects of 2D spiral and 3D radial sequences and has been proposed for $T_{1}$ mapping in static tissue but not dynamic cardiac tissue (110).

### 6.2. Motion estimation and correction

Current methods for motion-compensated $T_{1}$ mapping have a trade-off between data quantity and motion handling capacity. Acceleration factors increase as the temporal window of each bin gets narrower—respiratory motion-resolved imaging, for example, requires acceleration factors of 3–5 depending on the number of bins chosen. However, sparsity-based and low-rank reconstruction methods can leverage the correlations between bins to improve image quality in highly accelerated acquisitions. Nonrigid multiscale iterative registration methods can be used to correct motion between bins on different length scales, and averaging motion-corrected images can be used to reduce incoherent aliasing artifacts arising from undersampled acquisitions. Hybrid motion-corrected, motion-resolved approaches may help in isolating and correcting motion, allowing for lower acceleration factors and higher-quality maps. In particular, respiratory motion can be corrected and cardiac motion can be resolved, focusing on improving data quality about the more clinically relevant myocardial dynamics in the final data set. Additionally, better methods to detect and resolve cardiac motion are needed, particularly in patients with arrhythmias. Self-navigation or pilot-tone navigation may be able to accurately resolve irregular rhythms, and binning can be performed by RR interval length to divide data into different cardiac rhythm states corresponding to longer and shorter RR intervals (111).

Even within the same imaging framework, there is a trade-off between scan time, mapping regularization, and motion handling capacity: longer scans require less regularization but are susceptible to intra-scan, intra-bin motion due to inconsistent motion or hysteresis. Shorter scans may require highly accelerated, regularized reconstructions but have fewer motion components due to the reduced amount of cardiac or respiratory cycles in the whole scan. Cardiac-resolved techniques at high spatial resolution tend to lose sharpness across areas of the myocardium. Generally, strong edges such as the blood-myocardium border are preserved in undersampled reconstruction algorithms, but weaker edges such as boundaries between regions of dense fibrosis and myocardium can become blurred with over-regularized reconstructions. As well, multiparametric acquisitions tend to lose image sharpness when compared to single-parameter maps at the same scan time due to the increased number of contrasts to resolve multiple parameters simultaneously. Finally, a higher acquired spatial resolution may not herald improved image sharpness, as the effective resolution can be decreased if not enough high-order k-space data is acquired or if residual local motion blur persists after correction.

### 6.3. Sources of error in $T_{1}$ measurements

Technical improvements to $T_{1}$ mapping acquisition and reconstruction allow for robustness to confounding factors that affect $T_{1}$ mapping quality. In particular, data from multiple echo times and/or multiple flip angles can be used to generate maps of $B_{0}$ and $B_{1}$, respectively. Joint $T_{1}$ mapping and magnetic field mapping can correct for artifacts in the $T_{1}$ maps generated by static and transmitted field inhomogeneities. Additionally, work to jointly image cardiac motion and generate $T_{1}$ maps allows for cardiac motion-robust scans that incorporate functional information that removes the need for external cine scans. Similarly, respiratory motion correction during breath-holds and free-breathing scans allows for improved robustness and fewer re-acquired scans. Work in combining different preparations of $T_{1}$ contrast allows for reduced errors due to underlying limitations of preparation pulses (112).

When considering clinical standard methods, IR-based methods (e.g., MOLLI) tend to be more precise, whereas SR-based methods (e.g., SASHA) are more accurate. However, both approaches require motion correction to be more robust to residual cardiac and respiratory motion. Spatial denoising such as weighted total variation denoising or spatiotemporal denoising such as low-rank patch-based denoising can improve $T_{1}$ precision and reproducibility by suppressing statistical noise (113, 114). Dictionary-based methods can improve accuracy and reproducibility by using prior knowledge of imaging physics to simulate interactions between confounding factors and $T_{1}$ recovery.

### 6.4. Robust physics-based $T_{1}$ estimation methods

The standard method of curve-fitting is effective in determining $T_{1}$ and is relatively robust to noise given the low number of free parameters; however, it is susceptible to systematic errors due to intra-scan confounding factors like $T_{2}$, $B_{0}$ or $B_{1}$ inhomogeneity. Dictionary-based $T_{1}$ estimation methods are more powerful than standard curve-fitting as physics-based simulations inform the signal intensity fingerprints matched in the analysis process. However, these approaches suffer from the “curse of dimensionality” due to the voxelwise nature of the calculations and the number of parameters that need to be simulated for each patient-specific dictionary. Compression can be introduced to stabilize parameter estimation in such high-dimensional data sets, and multi-scale methods can be used to more precisely initialize parameters and limit the search space using auxiliary low-resolution images (115, 116). To improve post-processing computation times for dictionary-matching reconstructions, deep learning models have been proposed to accelerate the dictionary generation steps and directly estimate parameters from the input images within milliseconds (37, 117). However, such methods are currently scanner- and sequence-specific, and may not take into account confounding factors like magnetization transfer among others that dictate institution-specific normative $T_{1}$ values.

As well, deep learning models have been proposed to facilitate rapid map generation from fewer data points. For instance, acquiring fewer TIs in a Look–Locker acquisition can speed up the scan times by a factor of 2–3, but may introduce variability in $T_{1}$ mapping. One network to improve map estimation in such circumstances is MyoMapNet, where clinically diagnostic myocardial $T_{1}$ maps can be generated from four $T_{1}w$ images in traditional MOLLI scans (118).

### 6.5. Clinical translation and validation

To enable motion-corrected $T_{1}$ and multiparametric mapping in widespread clinical practice, larger studies of diverse patient cohorts are needed. Notably, sensitivity to underlying disease and ability to localize abnormal tissue regions must be evaluated to establish clinical feasibility. Prospective multi-center longitudinal studies accurately relating results from motion-corrected (multi)parametric mapping techniques to clinical diagnosis and prognosis must be performed to build clinician confidence toward regular use of these novel techniques.

One clinical barrier to more widespread adoption of motion-corrected $T_{1}$ mapping is the need for offline reconstructions that take hours to complete. Preliminary work integrating custom reconstruction and motion-correction techniques into the scanner software has shown promise to improve clinical feasibility, and frameworks to stream raw data to a dedicated computation server and back allow for a user-friendly implementation of computationally intensive tasks such as compressed sensing reconstruction, image registration, and dictionary generation (67, 117, 119). This is crucial for larger studies evaluating the diagnostic capabilities of novel motion-compensated sequences from the perspectives of patient comfort and clinical confidence.

One potential area of development in motion-corrected $T_{1}$ mapping is improved free-breathing $T_{1}$ mapping in the presence of cardiac implantable electronic devices such as implantable cardioverter-defibrillators and pacemakers. Patients with these devices would often benefit from improved characterization of associated cardiac pathophysiologies; however, this cohort rarely undergoes MR scans at present due to many difficulties in achieving high quality images. Methods exist to conduct 2D, breath-held, ECG-gated $T_{1}$ mapping in the presence of devices using wideband inversion pulses, GRE readouts, and $B_{0}$ correction (30, 120, 121). Additionally, preliminary data shows the potential for multiparametric mapping in the presence of devices (122). Incorporating 3D, free-breathing acquisitions into device-friendly protocols would improve patient comfort and expand utilization, as device patients may struggle to hold their breath repeatedly. More generally, shorter exam times would minimize risk due to time spent in the restrictive MR environment.

Most work on parametric and multiparametric mapping focuses on native $T_{1}$ measurements, although post-contrast $T_{1}$ mapping provides additional information in localizing regions of dense and diffuse fibrosis. Evaluating the capacity for motion-corrected (multi)parametric mapping techniques to resolve fine-scale features in complex arrhythmogenic substrate will be needed to move the field towards a quantitative approach to fibrosis characterization. Moving forward, we can utilize our knowledge of $T_{1}$ values (and values for other parameters) for normal tissue and various pathologies to generate maps of the underlying pathologies from the input (multiparametric) data. To this end, synthetic LGE images can be generated from post-contrast $T_{1}$ maps, and synthetic multi-contrast LGE images can be generated from co-registered post-contrast $T_{1}$ and $T_{2}$ maps (86, 123, 124).

To extend the clinical interpretation of multiparametric maps, tissue pathologies can be directly resolved from multiparametric data: current work in quantitative neuroimaging can use subvoxel analysis techniques to map white matter and gray matter concentration, and we hope similar results are achievable in the heart (125). Alternatively, tissue classification could be performed at the data (fingerprint) level without determining the fitting parameters—tissue classification models trained on the fingerprints have been shown to outperform those trained on the tissue parameters (126). Extending advanced multiparametric analysis techniques to more widespread use in cardiac MRI could simplify the reporting needed to diagnose pathologies.

2D and anisotropic 3D sequences often suffer from the need for a technologist to manually prescribe the correct oblique scan plane, usually the short axis. To mitigate this problem, automated scan planning using artificial intelligence has been proposed to find all relevant scan planes in less than a minute with one free-breathing acquisition. Alternatively, isotropic $T_{1}$ maps planned in the coronal, axial, or sagittal planes can be retrospectively reformatted to arbitrary imaging planes due to the high in-plane and through-plane resolution, removing the need for complicated scan planning.

## 7. Conclusion

In conclusion, motion-informed $T_{1}$ mapping methods allow for improved patient comfort, increased clinical utility, and higher-quality $T_{1}$ maps. Consideration of repeatable and non-repeatable confounding factors can improve $T_{1}$ mapping accuracy, precision, and reproducibility.
